# TRF2 couples muscle stem cell identity to regenerative repair

**DOI:** 10.1126/sciadv.aei7316

**Published:** 2026-07-31

**Authors:** Ji-Hyung Lee, Kiran Kumar Nakka, Ryan P. Calhoun, Sarah Hachmer, Adity Gupta, Eric Arreza, Lynn A. Megeney, Patrick Seale, Roger A. Greenberg, F. Jeffrey Dilworth, Foteini Mourkioti

**Affiliations:** ^1^ Department of Orthopaedic Surgery, Perelman School of Medicine, University of Pennsylvania, Philadelphia, PA 19104, USA.; ^2^ Sprott Center for Stem Cell Research, Regenerative Medicine Program, Ottawa Hospital Research Institute, Ottawa, ON, Canada.; ^3^ Department of Cellular and Molecular Medicine, University of Ottawa, Ottawa, ON, Canada.; ^4^ Department of Physiology and Cell Biology, Davis Heart and Lung Research Institute, College Medicine, The Ohio State University Wexner Medical Center, Columbus, OH 43210, USA.; ^5^ Department of Cell and Developmental Biology, Perelman School of Medicine, University of Pennsylvania, Philadelphia, PA 19104, USA.; ^6^ Institute for Diabetes, Obesity and Metabolism, Perelman School of Medicine, University of Pennsylvania, Philadelphia, PA 19104, USA.; ^7^ Department of Cell and Regenerative Biology, University of Wisconsin-Madison, Madison, WI 53706, USA.; ^8^ Department of Cancer Biology, Penn Center for Genome Integrity, Basser Center for BRCA, Perelman School of Medicine, University of Pennsylvania, Philadelphia, PA 19104, USA.; ^9^ Institute for Regenerative Medicine, Musculoskeletal Program, Perelman School of Medicine, University of Pennsylvania, Philadelphia, PA 19104, USA.

## Abstract

Stem cell–mediated regeneration is essential for tissue integrity. In skeletal muscle, tissue repair largely depends on muscle stem cells (MuSCs), which undergo dynamic cell-state transitions through making precise fate decisions during regeneration. However, the molecular regulators of cell-state conversion in MuSCs remain unclear. Here, we identify a previously unrecognized, noncanonical role for TRF2 in MuSC biology. TRF2 is dynamically regulated upon injury and required to preserve stem cell identity, support reparative myogenesis, and sustain self-renewal. MuSC-specific TRF2 disruption exacerbates muscular dystrophy pathology in mice, recapitulating key features of human disease. Mechanistically, TRF2 associates with regulatory regions enriched for DNA G-quadruplex–forming sequences at lineage-specific genes, sustaining their expression. These findings establish TRF2 as a pivotal regulator of adult stem cell function and tissue-specific regenerative responses.

## Teaser

Beyond telomere protection, TRF2 couples stem cell identity to regenerative repair through lineage-specific gene regulation.

## INTRODUCTION

Tissue restoration is essential to maintain skeletal muscles after injury. Skeletal muscle regeneration is exclusively achieved by the bona fide resident stem cells known as muscle stem cells (MuSCs), also called satellite cells (*1*). These cells are distinct from other cell types and reside between the sarcolemma and the basal lamina in a quiescent, low metabolic state (*2*). External cues, such as muscle trauma, activate the quiescent MuSCs to undergo rapid expansion and differentiation to reconstruct damaged muscle. During regeneration, a subset of activated MuSCs return to quiescence to replenish the stem cell reservoir, maintaining their quiescent status in homeostatic conditions (*1*). Yet, aging, diabetes, obesity, and several muscle diseases, including muscular dystrophies, are associated with an impaired MuSC function and a substantial decline in skeletal muscle regenerative capacity (*3*). Because MuSCs are essential for muscle maintenance and growth throughout life, exploiting the critical molecular regulators of these cells is vital to harness their remarkable potential for preserving skeletal muscles.

Quiescent MuSCs have been shown to express several molecular markers, including cell surface receptors, adhesion molecules, and transcription factors (*4*). Yet, the regulatory mechanism underlying these marker expressions and the establishment of the quiescent state is still unclear. MuSCs’ ability to undertake different fate choices depends on defined transcriptional hierarchies, posttranslational modification, and epigenetic events that ensure the proper spatiotemporal regulation of distinct genes in quiescence, activation, and differentiation (*4*). The capacity to transition between these different states is critical for achieving successful skeletal muscle repair in the context of disease, physiological aging, or injury-related damage (*5*, *6*). Yet, the cell-autonomous mechanisms through which stem cells maintain, exit, and reestablish their quiescence remain elusive. Moreover, the precise molecular signatures required for MuSCs to transition from quiescence toward reparative myogenesis remain largely unknown.

In this study, we identified a previously unrecognized stem cell function for Telomeric Repeat-binding Factor 2 (TRF2) in preserving MuSC stemness and regulating their regenerative capacity upon muscle damage. Furthermore, we demonstrated that TRF2 reduction in MuSCs from dystrophic mice, a mouse model for the fatal human Duchenne muscular dystrophy (DMD) (*7*), reduces life span and exacerbates disease progression, mirroring the symptoms observed in human patients. Contrary to other adult stem cells (*8*–*11*), we discovered that MuSC response to TRF2 deficiency is independent of telomere dysfunction. Instead, we uncovered that, mechanistically, TRF2 associates with regulatory regions enriched for G-quadruplex (G4)–forming DNA sequences at pivotal stem cell genes, supporting their transcriptional output and progression toward reparative myogenesis. Overall, these data offer valuable insights into the mechanism that governs stem cells in skeletal muscles and reveal a previously unknown noncanonical telomeric role for TRF2 as a nodal regulator of MuSC identity, maintenance, and functionality.

## RESULTS

### TRF2 expression changes dynamically in MuSCs following injury

**Fig. 1.**
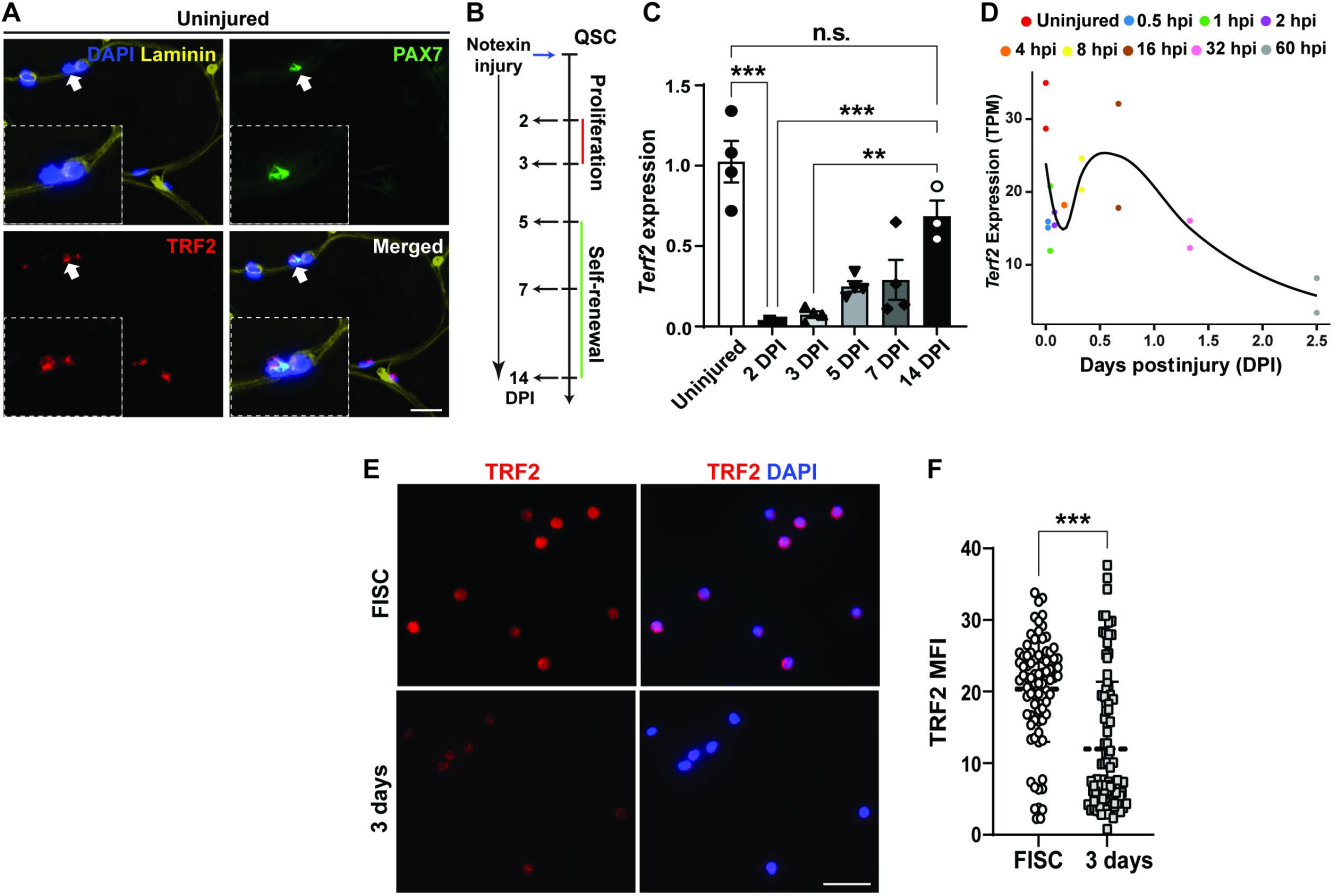
TRF2 is dynamically regulated in MuSCs during regeneration. (**A**) Representative immunofluorescence staining of laminin, Pax7, TRF2, and 4′,6-diamidino-2-phenylindole (DAPI; nuclei) of uninjured tibialis anterior (TA) muscle cryosections. Scale bar, 5 μm. (**B**) Schematic representation of MuSC isolation from TA muscle at uninjured [quiescence MuSCs (QSCs)] and at 2, 3, 5, 7, and 14 days post–notexin injury (DPI). (**C**) Gene expression analysis of *Terf2* (the gene encoding TRF2) from MuSCs isolated at the indicated time points after injury. (**D**) *Terf2* expression analysis in a public dataset (GSE189074) from MuSCs isolated from uninjured, 0.5, 1, 2, 4, 8, 16, 32, and 60 hours postinjury (hpi; *n* = 2 per groups). The *x* axis shows DPI. The *y* axis shows gene expression in transcripts per million (TPM). (**E**) Representative immunofluorescence images of TRF2 and DAPI (nuclei) in either freshly isolated cells (FISCs) or MuSCs maintained in culture for 3 days. Scale bar, 20 μm. (**F**) Quantification of TRF2 mean fluorescence intensity (MFI). *n* = 100 cells from four independent biological replicates. Data are presented as means ± SEM. Statistical analysis was performed using one-way analysis of variance (ANOVA) with Tukey’s multiple comparisons test in (C) and a two-tailed unpaired *t* test with Welch’s correction in (F). **P < 0.01; ***P < 0.001; n.s., not significant.

We first assessed the expression of TRF2 in skeletal muscles and found that it is highly expressed in quiescent MuSCs, as shown by its colocalization with the stem cell marker Pax7 (Fig. 1A). Because TRF2 can be transcriptionally regulated in a context-dependent manner (*12*), we examined *Terf2* expression (the gene encoding for the TRF2 protein) across distinct stem cell states. Live MuSCs were isolated using conventional isolation protocols (fig. S1A) from uninjured skeletal muscles as well as from muscles at defined time points postinjury (Fig. 1B). Quantitative reverse transcription polymerase chain reaction (qRT-PCR) analysis revealed that *Terf2* expression was sharply reduced in MuSCs isolated from 2 and 3 days postinjury (DPI) (Fig. 1C), consistent with a rapid transcriptional adjustment as MuSCs exit quiescence and enter proliferation, as a response to acute muscle damage. By 5 DPI, *Terf2* transcription gradually increased and reached comparable levels with uninjured MuSCs by 14 DPI (Fig. 1C), implying that *Terf2* expression is restored as MuSCs return to a quiescent, self-renewing state within their niche. Analysis of published RNA sequencing (RNA-seq) datasets at early time points postinjury (*13*), independently confirmed *Terf2* down-regulation in nonquiescent MuSCs by 2 DPI (Fig. 1D). Notably, while other telomere-associated proteins (also known as shelterins) were broadly down-regulated in MuSCs located within regenerating muscle tissue (fig. S1, B and C), *Terf2* was uniquely reexpressed in self-renewing MuSCs (Fig. 1C). Consistent with the gene expression data, TRF2 protein levels were significantly reduced in 3-day postisolated MuSCs compared with those in freshly isolated MuSCs (FISCs) (Fig. 1, E and F). This suggests that TRF2 is highly expressed in quiescent MuSCs and down-regulated in proliferating MuSCs. Together, these findings demonstrate that TRF2 undergoes dynamic regulation across distinct stem cell states in skeletal muscles.

### TRF2 is essential to sustain stem cell population and operates independently of cell death regulation

**Fig. 2.**
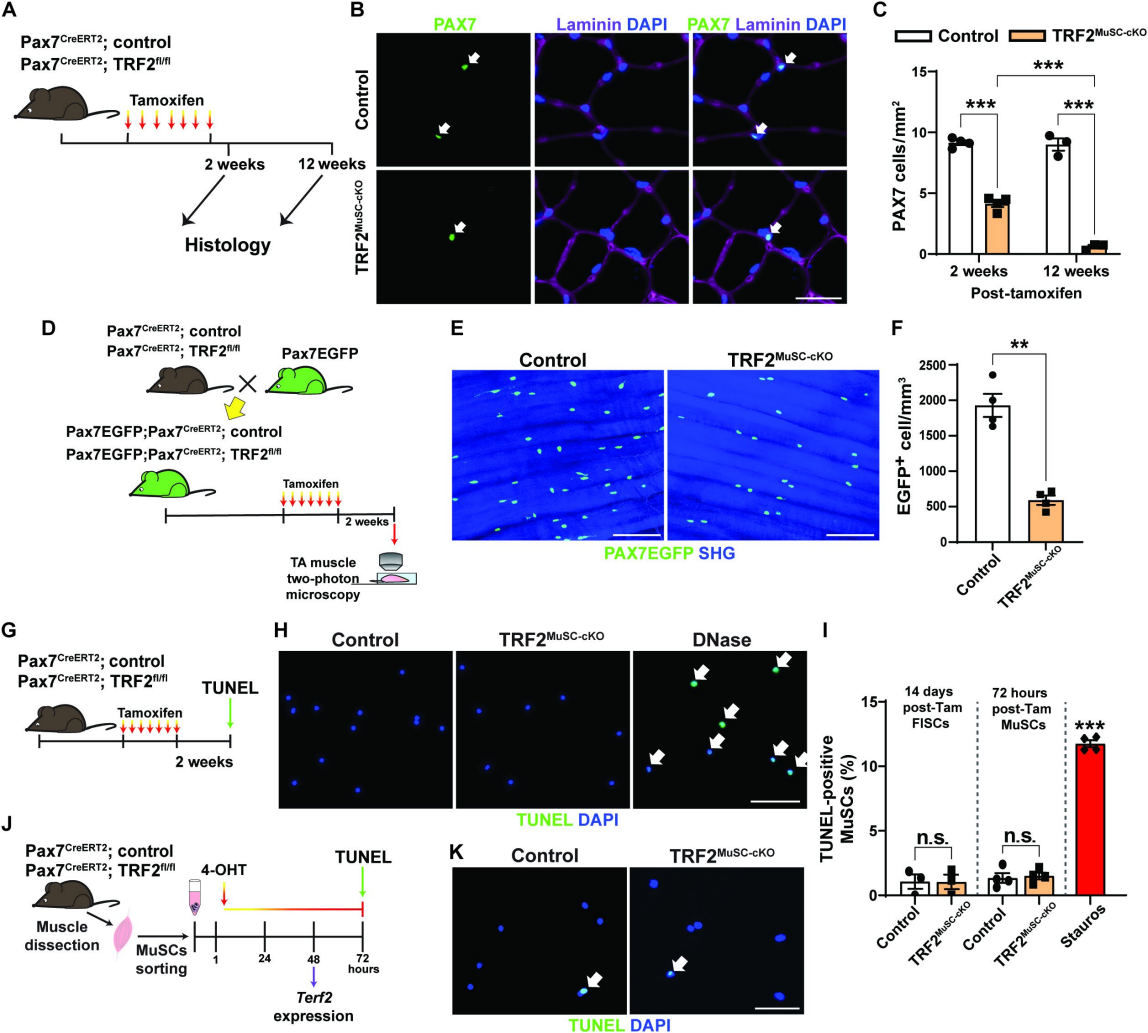
TRF2 is essential to sustain stem cells and acts independently of cell death. (**A**) Experimental scheme for tamoxifen (Tam)–induced deletion of TRF2 in MuSCs. (**B**) Representative immunofluorescence images of Pax7, laminin, and DAPI in TA muscle sections from control and TRF2^MuSC-cKO^ mice. White arrows indicate Pax7^+^ cells. Scale bar, 20 μm. (**C**) Quantification of Pax7^+^ cells normalized to fiber area at 2 and 12 weeks after Tam administration. (**D**) Breeding strategy used to generate Pax7EGFP/TRF2^MuSC-cKO^ mice. (**E**) Representative two-photon microscopy images showing Pax7EGFP and second harmonic generation (SHG) signals in controls and TRF2^MuSC-cKO^ muscles. Scale bars, 100 μm. (**F**) Quantification of EGFP^+^ cells per muscle volume (*n* = 4 mice per genotype). (**G**) Experimental scheme for terminal deoxynucleotidyl transferase–mediated deoxyuridine triphosphate nick end labeling (TUNEL) analysis of FISCs. (**H**) Representative TUNEL and DAPI staining of control and TRF2^MuSC-cKO^ FISCs. Deoxyribonuclease (DNase)–treated cells served as positive controls. White arrows indicate TUNEL-positive cells. Scale bar, 50 μm. (**I**) Quantification analysis of the % TUNEL-positive MuSCs. The left graphs are from FISCs for the experiment shown in (H), while the middle graphs are for the experiment shown in (K). Staurosporine-treated cells served as positive controls (red). More than 100 cells from three to four independent biological replicates per genotype were analyzed. (**J**) Experimental scheme of 4-hydroxytamoxifen (4-OHT) treatment of isolated MuSCs followed by TUNEL analysis. (**K**) Representative TUNEL and DAPI staining of control and TRF2^MuSC-cKO^ cells MuSCs followed by TUNEL analysis. White arrows indicate TUNEL-positive cells. Scale bar, 20 μm. Data are presented as means ± SEM. Statistical analysis was performed using two-way ANOVA with Tukey’s multiple comparisons test in (C) and two-tailed unpaired *t* tests with Welch’s correction in (F) and (I). **P < 0.01; ***P < 0.001; n.s., not significant.

To investigate the function of TRF2 in MuSC regulation, TRF2-floxed mice were crossed with Pax7^CreERT2^ mice to generate inducible MuSC-specific knockout of TRF2 (referred hereafter as TRF2^MuSC-cKO^) (Fig. 2A). Tamoxifen (Tam) administration efficiently reduced *Terf2* levels in MuSCs, as evidenced by both gene expression analysis (fig. S2A) and protein analysis of isolated MuSCs (fig. S2, B and C). Body weight, muscle mass, and muscle cross-sectional area (CSA) were not affected in TRF2^MuSC-cKO^ mice under steady-state conditions (fig. S2, D to I), indicating that TRF2 deletion in MuSCs does not affect muscles in the absence of injury. Flow cytometry analysis using cell surface markers by two different protocols, either vascular cell adhesion molecule (VCAM)^+^/α7-integrin^+^ or CD34^+^/α7-integrin^+^, revealed a decrease in the number of MuSCs in uninjured TRF2^MuSC-cKO^ muscles (fig. S2, J to M). Immunofluorescence staining on muscle sections (Fig. 2B) corroborated fewer Pax7^+^ cells in TRF2^MuSC-cKO^ muscles at 2 weeks, which was further worsened at 12 weeks post–Tam induction (Fig. 2C). To precisely enumerate MuSCs and gain insights into their properties, we crossed the Pax7EGFP mouse, a dynamic stem cell line that we previously generated (*14*), with the TRF2^MuSC-cKO^ mice (Fig. 2D). Two-photon microscopy was used to image tibialis anterior (TA) muscles, allowing visualization of MuSCs per total tissue volume in their natural muscle environment. The number of MuSCs was significantly diminished in TRF2^MuSC-cKO^ muscles, indicating that TRF2 reduction leads to a decline of detectable enhanced green fluorescent protein (EGFP)^+^ MuSCs (Pax7^+^ cells per muscle volume), even in the absence of injury (Fig. 2, E and F). These findings suggest a decline of stem cells over time under homeostatic conditions. Furthermore, studies have shown that quiescent MuSCs have long cellular protrusions, indicative of their genuine stem cell state, and these protrusions are retracted postinjury as cells shift away from deep quiescence (*15*–*17*). Notably, we found a reduction in the number of MuSC protrusions in TRF2^MuSC-cKO^ TA muscles, as evidenced by their altered morphology (fig. S2N) and a shift toward cell subtypes with fewer protrusions (fig. S2O). These data suggest that TRF2 insufficiency perturbs the stem cell quiescent state, even in the absence of injury.

Because TRF2 deficiency has been linked to cellular apoptosis in other systems (*18*, *19*), we next investigated whether the reduced MuSC population in uninjured TRF2^MuSC-cKO^ muscles is a consequence of cell death. Dead cell analysis using 7-aminoactinomycin D (7-AAD) in freshly isolated cells illustrated that there was no increase in dead MuSCs within TRF2^MuSC-cKO^ muscles (fig. S3, A and B). To further assess programmed cell death, MuSCs were isolated and subjected to terminal deoxynucleotidyl transferase–mediated deoxyuridine triphosphate nick end labeling (TUNEL) staining to identify apoptotic induction (Fig. 2G). We found that the apoptotic TRF2^MuSC-cKO^ MuSCs were comparable to control MuSCs (Fig. 2, H and I). To rule out that the absence of detectable apoptosis in TRF2^MuSC-cKO^ MuSCs is a false negative due to the unrecognized or lost MuSCs between Tam injection into mice and the completion of the stem cell isolation, we isolated MuSCs from control and TRF2^MuSC-cKO^ mice without Tam administration, allowed them to adhere to plates, and then introduced medium containing 4-hydroxytamoxifen (4-OHT) for 72 hours (Fig. 2J). After confirming that this method leads to efficient abrogation of *Terf2* expression (fig. S3C), TUNEL staining was performed to evaluate the extent of apoptosis (Fig. 2K). In agreement with the findings from FISCs, the apoptotic cell ratio of TRF2 knockout MuSCs at 72 hours postinduction was comparable with control MuSCs (Fig. 2I), supporting that the decreased MuSC number in TRF2^MuSC-cKO^ muscles is not a result of apoptosis. The coimmunostaining of PAX7/cleaved caspase-3 (CC3) on MuSCs (fig. S3D) further substantiated the absence of apoptosis, exhibiting less than 1% of PAX7^+^/CC3^+^ cells in both control and TRF2^MuSC-cKO^ samples (fig. S3E). Last, TRF2 deficiency has been associated with autophagic cell death in other cellular contexts (*20*, *21*). However, induction of autophagy or autophagosome formation was not increased in TRF2^MuSC-cKO^ MuSCs compared with that in controls (fig. S3, F to I). Collectively, these data demonstrate that TRF2 ablation disturbs stem cell quiescence and reduces MuSCs number even under steady-state conditions, without triggering cell death across the cell-death modality tested.

### MuSC-specific deletion of TRF2 severely impairs skeletal muscle regeneration after injury

**Fig. 3.**
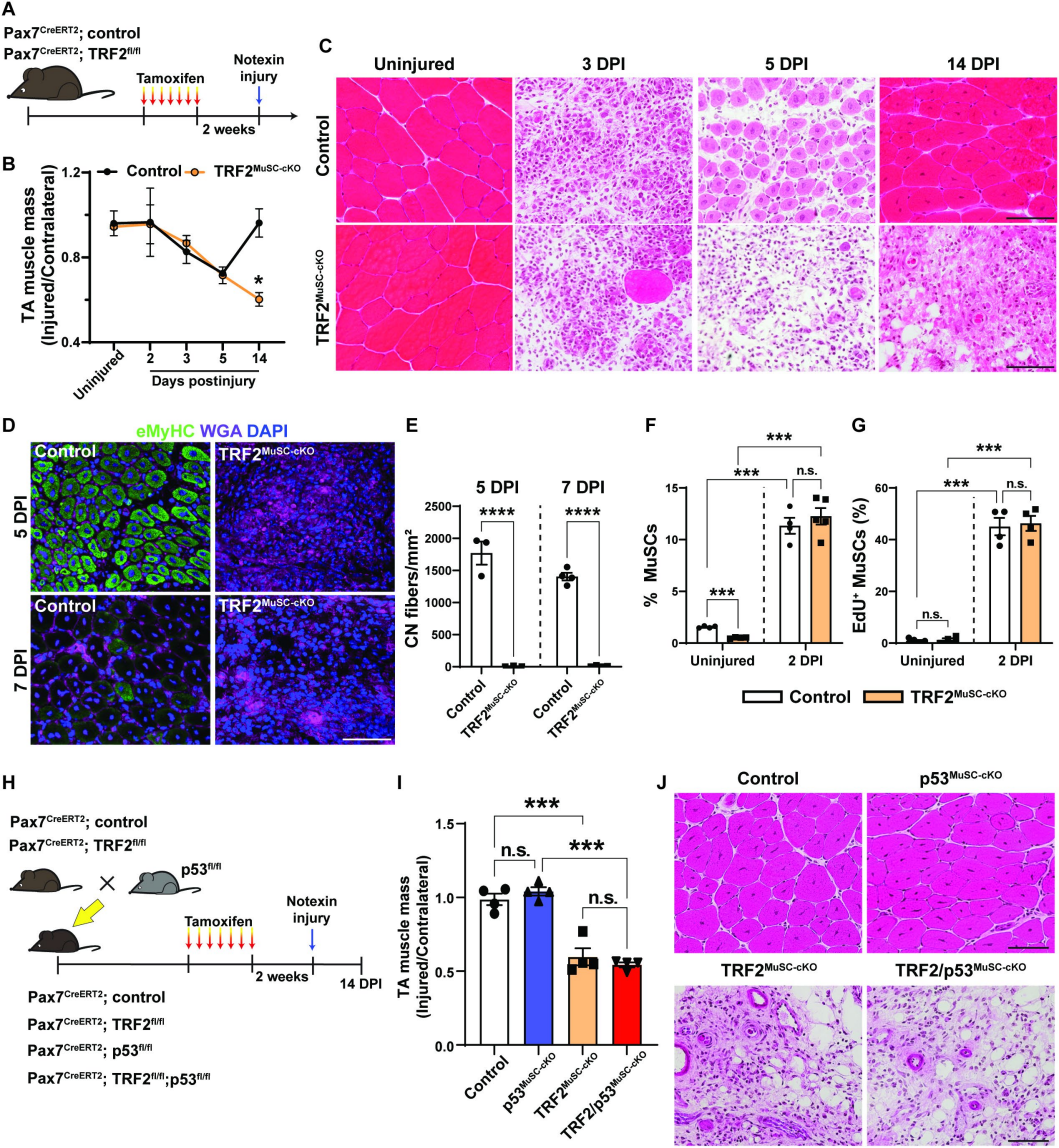
MuSC-specific deletion of TRF2 exhibit severe regeneration defects upon injury. (**A**) Experimental design used to access regenerative capacity in control and TRF2^MuSC-cKO^ muscles following notexin injury. (**B**) TA muscle mass measured at the indicated DPI (*n* = 4 mice per genotype). (**C**) Representative hematoxylin and eosin (H&E) staining of TA muscles from control (top) and TRF2^MuSC-cKO^ (bottom) mice at the indicated DPIs. Scale bars, 50 μm. (**D**) Representative images of newly forming myofibers stained for embryonic myosin heavy chain (eMyHC), nuclei (DAPI), and muscle fiber outlines [wheat germ agglutinin (WGA)] in control and TRF2^MuSC-cKO^ muscles at 5 DPI (top) and 7 DPI (bottom). Scale bar, 50 μm. (**E**) Quantification of centrally nucleated (CN) fibers per square millimeter in regenerating control and TRF2^MuSC-cKO^ muscles at 5 and 7 DPI (*n* = 3 to 4 mice per genotype). (**F**) Percentage of MuSCs in uninjured and 2 DPI muscles and (**G**) percentage of EdU-positive MuSCs (*n* = 4 to 5 mice per group). (**H**) Breeding strategy used to generate control, p53^MuSC-cKO^, TRF2^MuSC-cKO^, and TRF2/p53^MuSC-cKO^ double-mutant mice. Tam administration was initiated at 2 months of age. (**I**) TA muscle mass measured at 14 DPI in the indicated genotypes (*n* = 4 mice per genotype). (**J**) Representative H&E staining of TA muscles at 14 DPI. TRF2^MuSC-cKO^ and p53/TRF2^MuSC-cKO^ muscles exhibit similarly impaired regenerative morphology. Scale bars, 50 μm. Data are presented as means ± SEM. Statistical analysis was performed using two-way ANOVA with Tukey’s multiple comparisons test in [(B), (E), and (I)] and multiple two-tailed unpaired *t* test with Welch’s correction in [(F) and (G)]. *P < 0.05; ***P < 0.001; ****P < 0.0001; n.s., not significant.

Because adult MuSCs play a crucial role in repairing muscles after injury (*2*, *22*), we next sought to identify the impact of MuSC-specific TRF2 deletion on skeletal muscle regeneration. To achieve this, we injected notexin, a commonly used myotoxin snake venom, intramuscularly into the TA muscle of control and TRF2^MuSC-cKO^ mice to induce local necrosis (Fig. 3A). We found that muscle mass in TRF2^MuSC-cKO^ mice continued to decline after notexin injury, whereas control mice recovered within 14 DPI (Fig. 3B), indicating a marked impairment in muscle regeneration in TRF2^MuSC-cKO^ mice. Consistently, in contrast to control mice, which successfully reconstituted muscle structure by 14 DPI (Fig. 3C, top), TRF2^MuSC-cKO^ muscles lacked regenerating fibers (characterized by centralized nuclei) at both 5 and 14 DPI (Fig. 3C, bottom), indicating a profound regeneration defect. The absence of newly forming myofibers positive for the embryonic myosin heavy chain (eMyHC) marker at both early and later time points postinjury (Fig. 3, D and E) further supports the conclusion that loss of MuSC-specific TRF2 hinders the formation of differentiated fibers after injury and incapacitates myogenic commitment (fig. S4). In addition, severe tissue fibrosis was observed in TRF2^MuSC-cKO^ muscles at 14 DPI (fig. S5, A and B), suggesting substantial tissue damage and markedly impaired regeneration. We also observed adipose tissue accumulation at the injury site in TRF2^MuSC-cKO^ muscles at 14 DPI (fig. S5, C and D), another hallmark of defective muscle regeneration that resembles the previously reported phenotype of Pax7 knockout mice (*22*, *23*). We next asked whether the impaired muscle regeneration after injury is caused by a proliferation defect in TRF2^MuSC-cKO^ MuSCs. Notably, flow cytometry analysis of muscles at 2 DPI showed that TRF2^MuSC-cKO^ MuSCs expand comparably to control MuSCs (Fig. 3F). 5-ethynyl-2′-deoxyuridine (EdU) incorporation assays after injury likewise showed normal proliferative capacity of TRF2^MuSC-cKO^ MuSCs (Fig. 3G), corroborating the absence of proliferation defect in TRF2^MuSC-cKO^ MuSCs. Together, these data indicate that the regenerative failure caused by TRF2 loss is not due to defective MuSC expansion but instead arises from impaired progression through the myogenic program after injury.

p53 deletion has been reported to rescue the dysfunction of alveolar stem cells caused by TRF2 ablation (*8*). To investigate whether p53 deletion in MuSCs could alleviate the severe regeneration defects observed in injured TRF2^MuSC-cKO^ muscles (Fig. 3C), p53-floxed mice were crossed with Pax7^Cre/ERT2^ or TRF2^MuSC-cKO^ mice (referred to as p53^MuSC-cKO^ and TRF2/p53^MuSC-cKO^, respectively) (Fig. 3H and fig. S5E). We then assessed regeneration in p53^MuSC-cKO^ mice by measuring recovered muscle mass (Fig. 3I) and analyzing both the mean and distribution of fiber CSA (fig. S5, F and G). These experiments revealed that p53 depletion alone did not manifest any apparent defect in muscle repair (Fig. 3, I and J; and fig. S5, F to H). p53 deletion in TRF2^MuSC-cKO^ MuSCs was insufficient to restore either the abnormal muscle morphology (Fig. 3J and fig. S5I) or the fat accumulation observed in injured TRF2^MuSC-cKO^ muscles (fig. S5, H and J). These in vivo data further emphasize that, unlike in some other cell types (*8*, *24*), the requirement for TRF2 in MuSCs is not rescued by p53 loss. Collectively, these findings demonstrate that TRF2 expression in stem cells is indispensable for adult muscle regeneration and imply that TRF2 has a distinct function during MuSC-mediated myogenesis.

### TRF2 is required to maintain MuSC stem cell identity, support myogenic commitment, and preserve self-renewal capacity

**Fig. 4.**
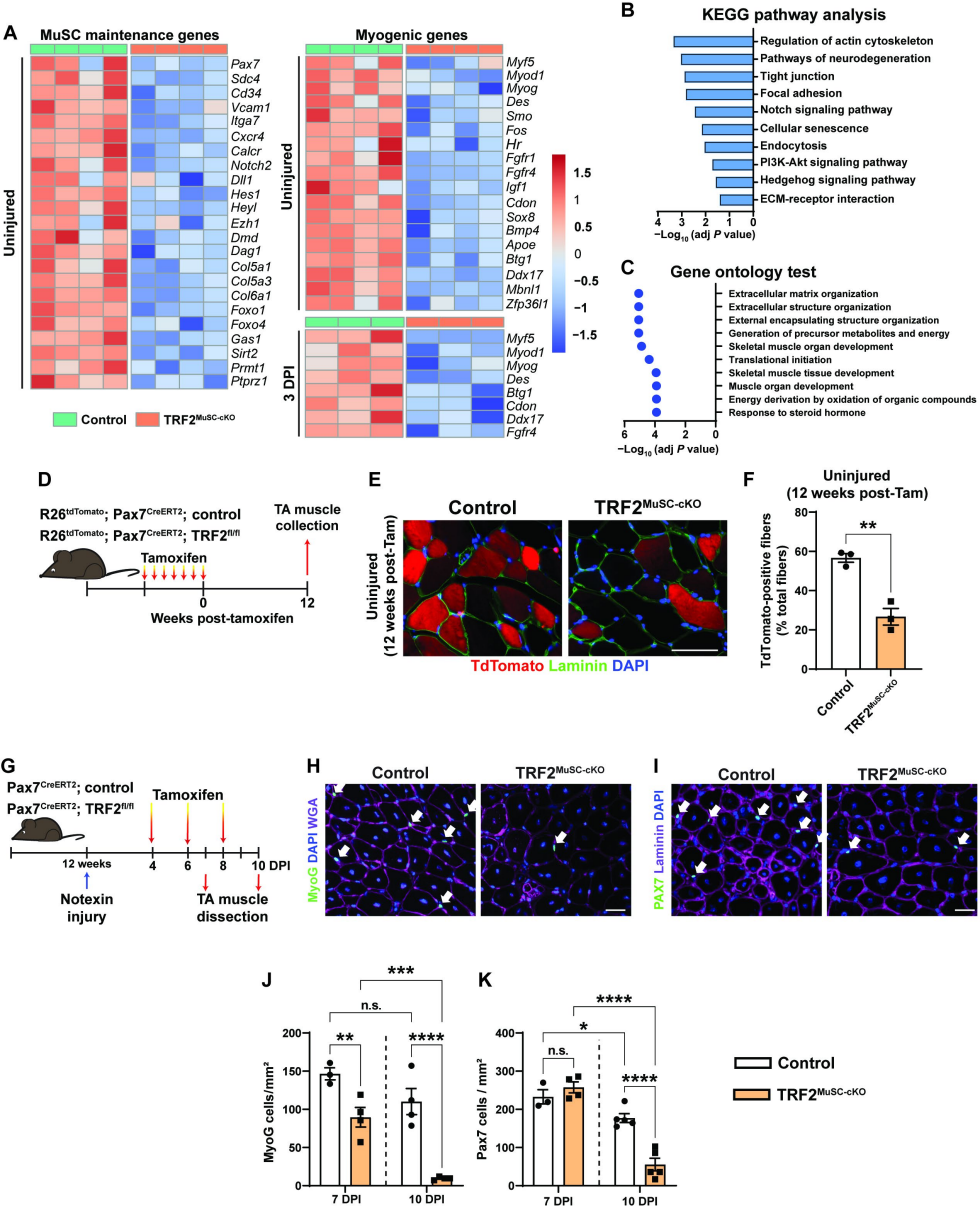
TRF2 preserves MuSC stem cell identity and maintains myogenic commitment and self-renewal. (**A**) Heatmaps showing genes involved in MuSC maintenance and myogenic genes. Differentially expressed genes (DEGs; adjusted *P* < 0.02, fold change of >1.5) in TRF2^MuSC-cKO^ MuSCs relatively to controls are displayed. Color scale represents relative expression (*z*-score) for each marker gene. (**B**) Top enriched pathways identified by Enrichr analysis using the “KEGG_2021_Human” gene-set library. ECM, extracellular matrix; PI3K, phosphatidylinositol 3-kinase. (**C**) Gene Ontology (GO) enrichment analysis of DEGs in uninjured TRF2^MuSC-cKO^ MuSCs compared with those in control. (**D**) Experimental scheme for lineage tracing in control and TRF2-deficient Pax7-lineage mice carrying the *R26*^tdTomato^ reporter. (**E**) Representative immunofluorescence images of uninjured TA muscles collected at 12 weeks post–Tam administration and stained for tdTomato, laminin, and DAPI. Scale bar, 50 μm (**F**) Quantification of tdTomato^**+**^ fibers as a percentage of total myofibers in control and TRF2^MuSC-cKO^ muscles. (**G**) Experimental design used to access MuSC self-renewal following injury. (**H**) Representative TA muscle sections stained for MyoG, DAPI, and WGA in control and TRF2^MuSC-cKO^ mice. Scale bar, 20 μm. (**I**) Representative TA muscle sections stained for Pax7, DAPI, and WGA in control and TRF2^MuSC-cKO^ mice. Scale bar, 20 μm. (**J**) Quantification of MyoG^+^ cells per square millimeter at 7 and 10 DPI (*n* = 4 mice per genotype). (**K**) Quantification of Pax7^+^ cells per square millimeter at 7 and 10 DPI (*n* = 4 mice per genotype). Data are presented as means ± SEM. Statistical analysis was performed using two-tailed unpaired *t* test with Welch’s correction in (F) and two-way ANOVA with Tukey’s multiple comparisons test in (J) and (K). *P < 0.05; **P < 0.01; ***P < 0.001; ****P < 0.0001; n.s., not significant.

Defective MuSC function has been linked to impaired expression of stem cell markers (*25*). To define the molecular features of TRF2-deficient MuSCs, we conducted transcriptomic analysis. This analysis revealed minimal variability among intergroup replicates but significant variability (more than 50%) between groups (fig. S6A), indicating marked transcriptional differences between control and mutant MuSCs. We identified 837 up-regulated and 1383 down-regulated genes in isolated TRF2^MuSC-cKO^ MuSCs relative to controls (fig. S6B). Notably, genes involved in stem cell maintenance and/or self-renewal were globally down-regulated in TRF2^MuSC-cKO^ MuSCs (Fig. 4A). These included known stem cell markers (*Pax7*, *Sdc4*, *CD34*, and *Cxcr4*), canonical Notch genes (*Notch2*, *Dll1*, *Hes1*, and *Heyl1*), noncanonical Notch targets [*Ezh1* (*26*) and *Calcr*, which is also a marker of quiescent MuSCs (*27*)], epigenetic regulators (*Sirt2* and *Prmt1*), and extracellular matrix proteins (*Col5a1*, *Col5a3*, and *Col6a1*). Additionally, myogenic factors known to be expressed in both quiescent and activated MuSCs, such as *Myf5* and *Myod1* (*28*–*32*), were also down-regulated (Fig. 4A). Pathways involved in stem cell niche interactions, including tight junctions, focal adhesions, and extracellular matrix–receptor interactions, were likewise down-regulated (Fig. 4B). These pathways are known to support stem cell maintenance and self-renewal (*33*, *34*). Unbiased Gene Ontology (GO) analysis further corroborated that genes associated with skeletal muscle development were significantly down-regulated (Fig. 4C and fig. S6, C and D), suggesting that TRF2^MuSC-cKO^ MuSCs lose their myogenic stem cell identity without evidence for activation of alternative lineage programs (fig. S6, C to F). Last, lineage-tracing analysis using TRF2^fl/fl^; R26^tdTomato^; Pax7^CreERT2^ mice, in which TRF2-deleted MuSCs are simultaneously labeled by tdTomato expression, ascertained that the fat accumulation in TRF2^MuSC-cKO^ muscle is not a consequence of trans-differentiation of MuSCs into adipogenic cells, as demonstrated by the lack of tdTomato expression in perilipin-positive adipocytes (fig. S6, G and H).

MuSCs has been reported to contribute to myofiber formation in sedentary conditions, leading to the appearance of tdTomato-labeled myofibers (*35*, *36*). We therefore asked whether the reduced transcriptomic quiescence signature observed in TRF2^MuSC-cKO^ MuSCs translates into functional quiescence impairment. Consistent with previous reports (*35*, *36*), in vivo lineage-tracing assays (Fig. 4D) revealed MuSC contribution to myofibers, as indicated by tdTomato^+^ myofibers (Fig. 4, E and F; and fig. S7, A and B). Intriguingly, TRF2^MuSC-cKO^ mice exhibited a significantly lower frequency of tdTomato^+^ myofibers (Fig. 4, E and F; and fig. S7, A and B), indicating impaired long-term maintenance and/or reduced contribution of TRF2-deficient MuSCs in uninjured muscle. To delineate the cellular mechanism underlying fewer tdTomato^+^ fibers in TRF2^MuSC-cKO^ muscle, we first investigate whether TRF2 ablation induces precocious activation of quiescent TRF2^MuSC-cKO^ MuSCs (fig. S7C). However, aberrant activation was not observed in TRF2^MuSC-cKO^ MuSCs, as evidenced by the comparable number of EdU^+^ MuSCs in control and TRF2^MuSC-cKO^ muscles (fig. S7, C to E). To further define the behavior and fate of TRF2-deficient MuSCs after injury, we examined their kinetics upon exiting from quiescence and assessed senescence. We found that TRF2 loss disrupts proper MuSC activation process but preserved proliferative capacity after activation (fig. S7, F to I), consistent with our in vivo proliferation assay (Fig. 3, F and G).

To explore the ability of TRF2^MuSC-cKO^ cells to return to their stem cell niche, we administrated Tam into postinjured mice during the period in which self-renewal normally takes place during muscle repair (Fig. 4G). We adopted this strategy because TRF2^MuSC-cKO^ muscles lacked regenerating myofibers (Fig. 3, D and E), precluding assessment of niche reentry through standard regenerative readouts. Consistent with the myogenic defect in TRF2^MuSC-cKO^ MuSCs, the number of MyoG^+^ cells, representing differentiated MuSC progeny, progressively declined in mutant muscles (Fig. 4, H and J). We also quantified Pax7^+^ cells located within the basal lamina at 7 and 10 DPI (Fig. 4, I to K). In control muscles, we observed fewer Pax7^+^ MuSCs within the basal lamina at 10 DPI than at 7 DPI (Fig. 4K), consistent with the idea that MuSCs progressively return to quiescence and reoccupy the niche during the later stages of regeneration, in line with previous in vivo studies showing that MuSCs reacquire quiescence after 7 DPI (*37*, *38*). Notably, although TRF2^MuSC-cKO^ muscles exhibited a similar number of Pax7^+^ MuSCs at 7 DPI, this population was significantly reduced by 10 DPI (Fig. 4, I and K). These findings suggest that TRF2-deficient MuSCs fail to maintain their stem cell pool because of defective niche reentry.

Recent studies have shown that MuSCs can undergo direct commitment under homeostatic condition (*39*, *40*). Because our data suggest that quiescent MuSCs do not spontaneously enter the cell cycle, we hypothesized that TRF2-deficient MuSCs instead undergo direct commitment because of an inability to return to quiescence. Notably, the proportion of EdU^+^/MyoG^+^ cells was decreased in TRF2^MuSC-cKO^ muscle at 7 DPI (fig. S8, B and D), whereas the proportion of EdU^+^/PAX7^+^ cells was unaltered (fig. S8, C and D). These results suggest that TRF2 ablation biases MuSCs toward reduced generation of committed myogenic daughters rather than cyclic commitment, abrogating the maintenance of the stem cell population. These findings demonstrate that the reduced tdTomato^+^ fibers in TRF2^MuSC-cKO^ muscle (Fig. 4, E and F) are attributed to a cumulative effect of both impaired long-term maintenance (a self-renewal defect) and reduced contribution of MuSCs to myofiber (a myogenic defect). Last, we also found no signs of increased cellular senescence in TRF2^MuSC-cKO^ MuSCs (fig. S9). Together, these results indicate that TRF2 depletion perturbs MuSC quiescence by selectively impairing activation dynamics without affecting the proliferative capacity of activated MuSCs or promoting senescence. Together, these findings identify TRF2 as a critical regulator of MuSC quiescence maintenance, activation, self-renewal, and both the efficiency and mode of myogenesis while leaving overall MuSC proliferative capacity largely intact.

### Blunted MuSC function in TRF2^MuSC-cKO^ mice is not attributable to telomeric DNA damage responses

Because TRF2 is known to protect telomeres, we investigated whether the severe MuSC defects in TRF2^MuSC-cKO^ MuSCs are associated with telomere dysfunction. TRF2 has been reported to regulate telomere length in various cell lines (*41*–*44*). Thus, we sought to address whether TRF2^MuSC-cKO^ MuSCs have defects in telomere length homeostasis by using the MuQ–fluorescence in situ hybridization (FISH) method (fig. S10A), which was previously optimized and validated specifically for measuring telomere length in scarce populations of delicate stem cells (*45*, *46*). Intriguingly, MuSC telomere length is comparable between controls and TRF2^MuSC-cKO^ cells, both in terms of mean telomere length (fig. S10, B and C) and telomere length distribution (fig. S10D), the latter of which also captures rare cell populations that may be obscured by average-basis analyses. These findings demonstrate that TRF2 is not an essential regulator of telomere length in quiescent MuSCs.

**Fig. 5.**
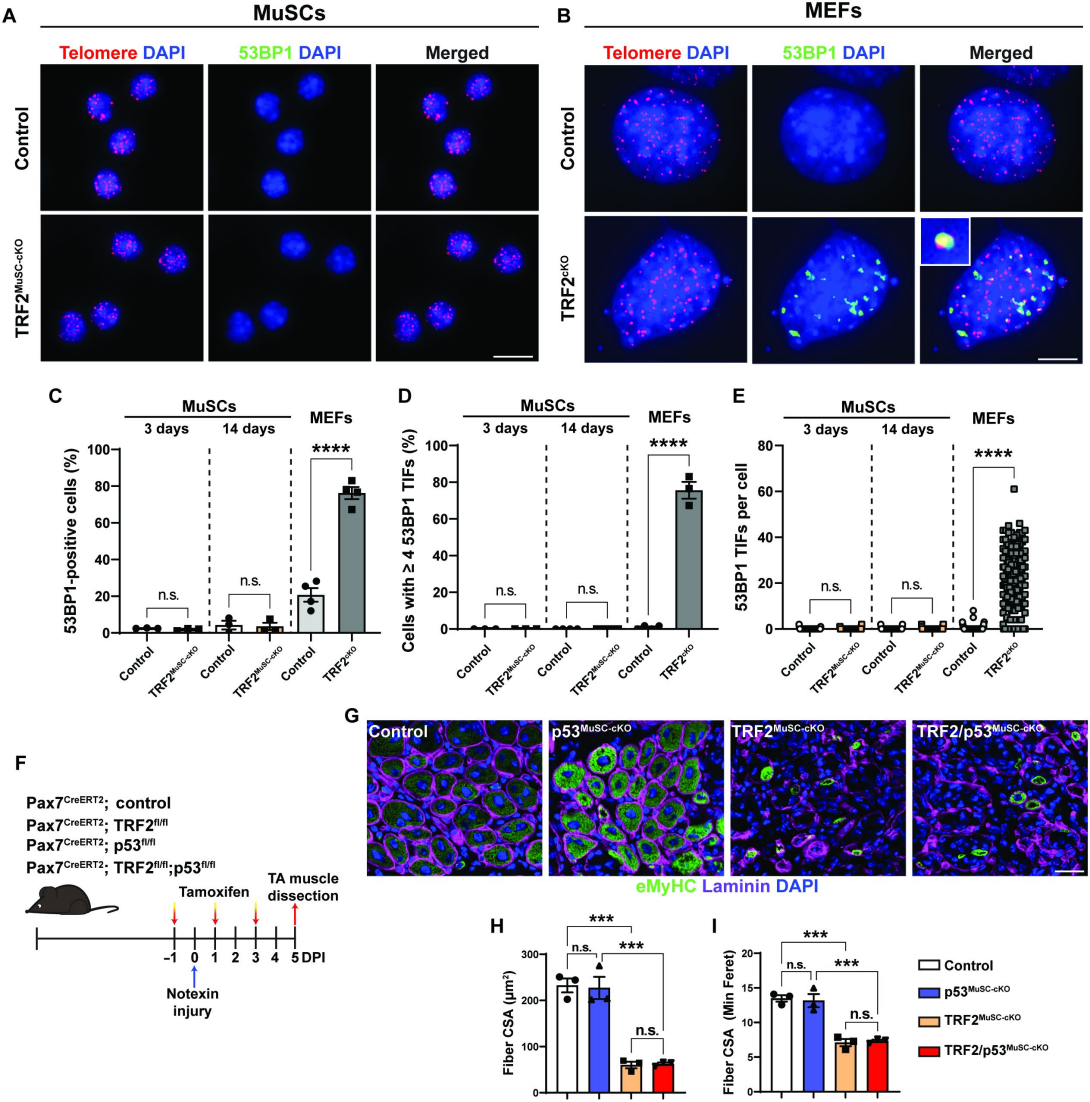
TRF2 deficiency in MuSCs does not induce substantial telomere dysfunction or a p53-dependent regenerative defect. (**A** and **B**) Representative analysis of telomere dysfunction by telomere fluorescence in situ hybridization (FISH) combined with 53BP1 immunostaining in MuSCs (A) and MEFs (B). MuSCs were analyzed 3 and 14 days after TRF2 deletion, whereas MEFs were analyzed at 3 days postinduction. Telomeres are shown in red, 53BP1 in green, and nuclei in DAPI (blue). Insets highlight representative telomere dysfunction-induced foci (TIFs). Scale bars, 5 μm. (**C** to **E**) Quantification of telomere-associated DNA damage, including the percentage of 53BP1-positive cells (C), the percentage of cells containing ≥4 TIFs (D), and the number of TIFs per cell (E). More than 100 cells were analyzed per cell type. (**F**) Experimental design used to access the impact of p53 deletion on TRF2^MuSC-cKO^ mice during the period of DDR activation. Tam administration was initiated at 3 months of age. (**G**) Representative images of newly forming myofibers stained for eMyHC, nuclei (DAPI), and myofiber boarders (laminin) in control, p53^MuSC-cKO^, TRF2^MuSC-cKO^, and TRF2/p53^MuSC-cKO^ muscles. Scale bar, 20 μm. (**H** and **I**) Quantification of regenerating myofiber size measured as CSA (H) and minimal Feret diameter (I). *n* = 3 mice per genotype. Data are presented as means ± SEM. For telomere dysfunction analyses, *n* = 3 mice per genotype were analyzed for control and TRF2^MuSC-cKO^ groups. Statistical analysis was performed using two-way ANOVA with Tukey’s multiple comparisons test in [(C) to (E)] and in [(H) and (I)]. **P < 0.01; ****P < 0.0001; n.s., not significant.

*TRF2* deficiency has previously been shown to initiate DNA damage response (DDR) at telomeres (*47*), typically appearing 24 to 96 hours postinduction and resolved at later time point in mouse embryonic fibroblast (MEF) cultures (*48*, *49*). To accommodate the time frame for telomeric DDR detection, we isolated MuSCs from control and TRF2^MuSC-cKO^ mice at 3 and 14 days postinduction and assessed DDR activation. The immunofluorescence (IF) staining of 53BP1, which is well-established DDR marker (*50*) as well as a classically observed in TRF2-deficient cells preceded telomere fusion (*44*, *51*), identified the absence of DDR activation in TRF2-deletd MuSCs at both 3 and 14 days postinduction (Fig. 5, A and C). Induction of telomeric DNA damage, also known as telomere-induced foci (TIFs), was not detected in TRF2^MuSC-cKO^ MuSCs, as assessed by telomere-FISH combined with 53BP1 (Fig. 5, A to E). This contrasts to TRF2-deleted MEF, where both global and telomeric 53BP1 signals were apparent (Fig. 5, B to E). We also measured phosphorylation of ataxia-telangiectasia mutated (ATM) kinase, an initial event in DDR activation following TRF2 deficiency (*51*, *52*). Intriguingly, ATM phosphorylation was comparable between control and TRF2^MuSC-cKO^ MuSCs (fig. S11, E to H), whereas it was elevated in TRF2-deleted MEFs (fig. S11, F to H). We next investigated additional well-established DDR markers, γH2A.X (*53*), and found that both global and telomeric γH2A.X levels were not affected in TRF2^MuSC-cKO^ MuSCs (fig. S11, I to M). In contrast, TRF2-deleted MEFs showed robust accumulation of γH2A.X TIFs (fig. S11, J to M). To further support the absence of telomeric DNA damage in TRF2^MuSC-cKO^ MuSCs, we isolated MuSCs without Tam administration, cultured them in medium containing 4-OHT for 60 hours (fig. S12A), and measured telomeric DNA damage. Again, both telomeric DDR (fig. S12, C, E, and F) and global DDR (fig. S12G) were comparable between control and TRF2^MuSC-cKO^ MuSCs. This contrasted with TRF2-deleted MEFs, in which robust TIFs formation and global DDR activation were evident at 60 hours after induction (fig. S12, B and D to G). We also observed aberrant chromosome structures, including micronuclei and chromosomal bridges, in TRF2-deleted MEFs, while these abnormalities were completely absent in TRF2^MuSC-cKO^ MuSCs (fig. S12, H and I). Last, analysis of telomere foci number revealed that control and TRF2^MuSC-CKO^ MuSCs have comparable numbers of telomeres, contrary to TRF2-deleted MEFs, in which telomere foci number was significantly reduced (fig. S12J). These findings do not support widespread telomere fusion or canonical chromosome-end dysfunction as the primary basis of the observed phenotype in TRF2-deleted MuSCs. Together, across the time points and conditions assayed, these data indicate that TRF2 deletion does not trigger telomeric DDR activation in MuSCs.

Last, telomere dysfunction and TRF2 deficiency are known to elicit cellular response via p53-mediated pathways (*18*, *50*). However, our data showed that p53 deletion did not restore the regeneration defects caused by TRF2 deficiency in MuSCs (Fig. 3, H to J; and fig. S5, H to J). In actively dividing cultured MEFs, TRF2 deletion triggers ATM/p53-dependent pathway between 36 and 120 hours after induction (*48*). To rule out the possibility that we might have missed the time window in which p53 could rescue the MuSC defects in our previous in vivo experiments (Fig. 3H), we induced TA muscle injury 1 day after Tam administration (Fig. 5F). This design allowed us to assess the regenerative capacity of TRF2^MuSC-cKO^ and TRF2/p53^MuSC-cKO^ mice shortly after the deletion of TRF2 and p53. This analysis further demonstrated that p53 deletion did not rescue the regeneration failure of TRF2^MuSC-cKO^ mice, as shown by the persistent severe reduction in both the numbers and size of newly formed fibers (Fig. 5, G to I).

Collectively, the comparable telomere length, absence of telomeric and global DDR activation, preservation of telomere foci numbers, and independence from the TRF2-p53 axis, together with normal proliferation rates (Fig. 3, F and G) and lack of increased senescence (fig. S9), indicate that the defects observed in TRF2-deficient MuSCs are unlikely to result from a conventional telomeric DDR pathway. Instead, these findings support a model in which TRF2 operates predominantly through an extra-telomeric mechanism in MuSCs during muscle regeneration.

### TRF2 dysregulation in stem cells aggravates disease progression in muscular dystrophy

**Fig. 6.**
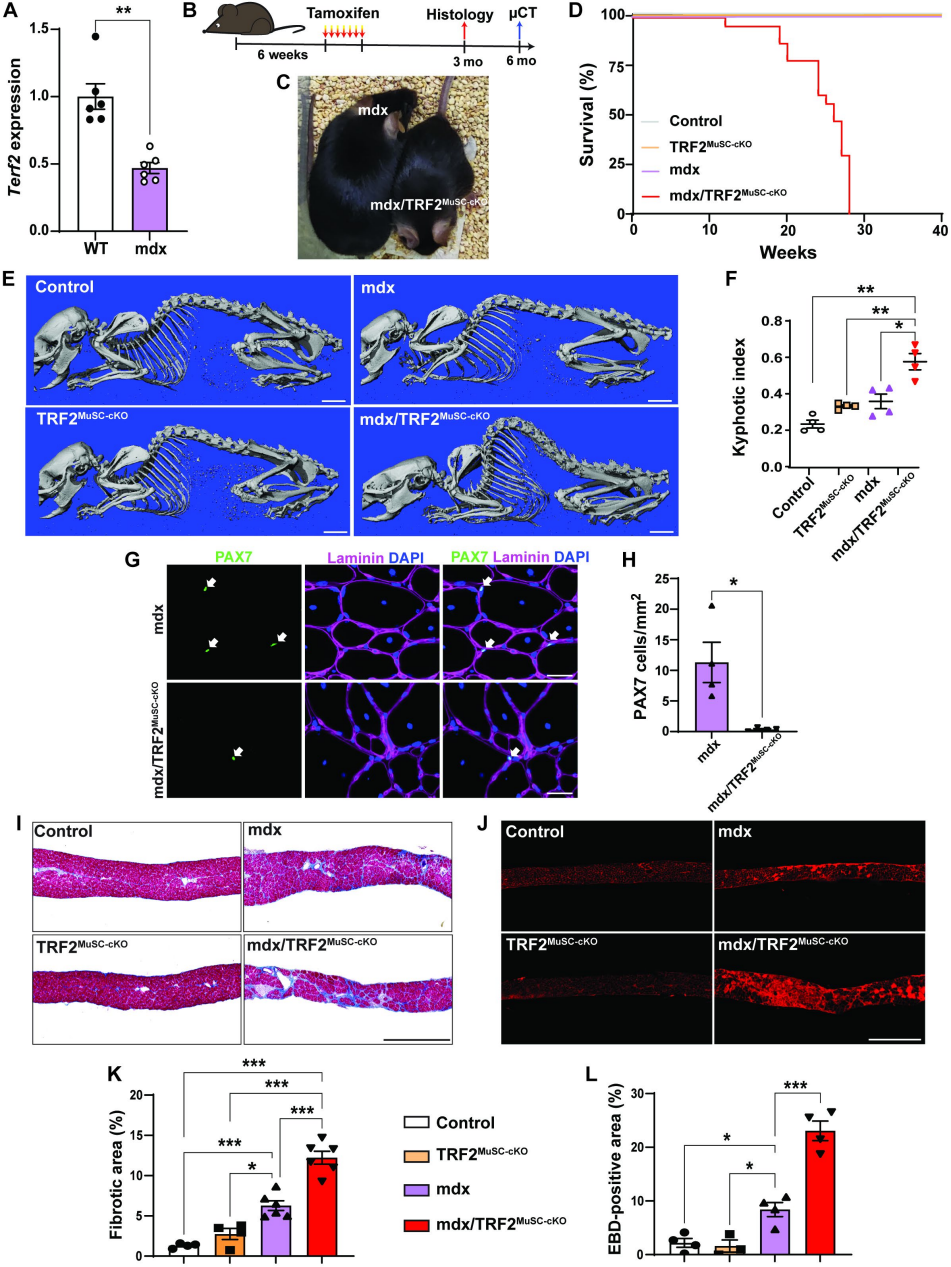
MuSC-specific TRF2 loss exacerbates disease progression and reduces survival in mdx mice. (**A**) *Terf2* expression in MuSCs isolated from control and dystrophic muscles (*n* = 6 mice per genotype). (**B**) Experimental scheme for MuSC-specific TRF2 deletion in mdx mice by Tam administration. mo, months. (**C**) Representative gross morphology of mdx and mdx/TRF2^MuSC-cKO^ mice at 20 weeks of age. (**D**) Kaplan-Meier survival analysis of control, TRF2^MuSC-cKO^, mdx, and mdx/TRF2^MuSC-cKO^ mice. *n* = 15 (control), *n* = 14 (TRF2^MuSC-cKO^), *n* = 21 (mdx), and *n* = 23 (mdx/TRF2^MuSC-cKO^). (**E**) Representative whole-body micro–computed tomography (μCT) images of control, TRF2^MuSC-cKO^, mdx, and mdx/TRF2^MuSC-cKO^ mice. Scale bars, 5 mm. (**F**) Quantification of kyphotic index from whole-body μCT images (*n* = 4 mice per genotype). (**G**) Representative immunofluorescence images of Pax7, DAPI, and laminin (basal lamina) in TA muscles from mdx and mdx/TRF2^MuSC-cKO^ mice. Scale bar, 20 μm. (**H**) Quantification of Pax7^+^ cells in TA muscles (*n* = 4 mice per genotype). (**I**) Representative Masson’s trichrome staining of diaphragm muscles. Scale bar, 500 μm. (**J**) Representative images of Evans blue dye (EBD) uptake as an indication of myofiber damage. Scale bar, 500 μm. (**K**) Quantification of fibrotic area (in blue) (*n* = 4 to 6 mice per genotype). (**L**) Quantification of EBD-positive fibers (*n* = 3 to 4 mice per genotype). Data are presented as means ± SEM. Statistical analysis was performed using two-tailed unpaired *t* test with Welch’s correction in [(A) and (H)] and two-way ANOVA with Tukey’s multiple comparisons test in [(F), (K), and (L)]. *P < 0.05; **P < 0.01; ***P < 0.001.

MuSC dysfunction is a characteristic feature of DMD, a devastating muscle degenerative X-linked genetic disease caused by a genetic mutation in the structural gene *dystrophin* (*7*). A progressive decline in stem cell population and flawed regenerative capacity has been reported in mdx mice (*54*, *55*), a mouse model of DMD that carries a similar dystrophin mutation as patients with DMD. Previous research suggests that, although DMD is initiated by dystrophin deficiency, it progresses as a stem cell disease (*54*, *55*). We therefore investigated TRF2 levels in diseased muscle and discovered that *Terf2* expression was significantly reduced in dystrophic MuSCs (Fig. 6A). In contrast, expression levels of the other two DNA-binding shelterin components, *Terf1* and *Pot1*, remained unchanged (fig. S13A). To determine the consequences of reduced TRF2 in muscular dystrophy, we crossed the conventional mdx^4cv^ mice with TRF2^MuSC-cKO^ mice to generate double-mutant mdx/TRF2^MuSC-cKO^ mice. Cre was activated at 6 weeks of age, and mice were analyzed at later time points (Fig. 6B). Macroscopic observation by 20 weeks of age already revealed that mdx/TRF2^MuSC-cKO^ mice were slightly smaller than controls and displayed a slack posture (Fig. 6C), suggesting the development of pathological features. Unlike the muscle hypertrophy typically observed at early stages in mdx muscles (*56*), mdx/TRF2^MuSC-cKO^ mice exhibited severe muscle atrophy (fig. S13B), consistent with observations in young adult patients with DMD (*7*). Further analysis showed significant reductions in body weight and skeletal muscle mass in mdx/TRF2^MuSC-cKO^ mice compared with those in mdx mice (fig. S13, C to F), further demonstrating that the loss in body weight was largely attributed to muscle loss. To examine survival, we conducted Kaplan-Meier analysis and found that mdx/TRF2^MuSC-cKO^ mice had a significantly shorter life span, with mortality beginning at 12 weeks of age. These mice do not survive beyond 28 weeks of age (Fig. 6D), in contrast to mdx mice that have a life span comparable to that of wild-type mice (*57*). In addition, micro–computed tomography (microCT) analysis revealed severe spinal deformities in mdx/TRF2^MuSC-cKO^ mice (Fig. 6, E and F), known as kyphosis, a hallmark of DMD in patients that is not reproduced in mdx mice (*57*). Because the MuSC number was diminished in TRF2^MuSC-cKO^ muscle (Fig. 2 and fig. S2) and self-renewal was impaired (Fig. 4K), we next examined MuSC abundance during chronic injury in mdx/TRF2^MuSC-cKO^ mice. We found a marked reduction in Pax7^+^ MuSCs, by ∼95%, in mdx/TRF2^MuSC-cKO^ muscles compared with that in mdx mice (Fig. 6, G and H). These findings suggest that loss of TRF2 in dystrophic MuSCs accelerates disease progression. Consistent with this idea, histopathological abnormalities (Fig. 6I) and the percentage of Evans blue dye (EBD)–positive fibers (Fig. 6J), a membrane damage indicator, were both augmented in diaphragm muscles (Fig. 6, K and L), which is the most affected muscles in DMD (*7*). Notably, diaphragm thickness has significantly reduced in mdx/TRF2^MuSC-cKO^ muscles compared with that in age-matched mdx muscles (fig. S13G), indicative of impaired respiratory function. Similarly, increased EBD positivity was observed in the gastrocnemius of mdx/TRF2^MuSC-cKO^ mice (fig. S12, H and I), further suggesting that muscle damage is overall worsened in these mice. Our previous studies showed that telomere attrition aggravates the progression of DMD (*46*, *55*). We therefore asked whether telomere shortening contributed to the worsened phenotype of mdx/TRF2^MuSC-cKO^ mice. Consistent with our earlier data (figs. S10 and S12J), both telomere length and telomere number were comparable in MuSCs isolated from mdx and mdx/TRF2^MuSC-cKO^ mice (fig. S13, J to M). Together, these findings demonstrate that TRF2 deficiency in dystrophic MuSCs exacerbates DMD progression, exhibiting many more clinical signs of patients with DMD, and further highlight the importance of identifying the molecular mechanism by which TRF2 reduction in MuSCs drives such an intense phenotype.

### Extra-telomeric TRF2 associates with regulatory regions enriched for putative G4-forming sequences at stem cell and early myogenic genes to maintain stem cell identity of the myogenic lineage

**Fig. 7.**
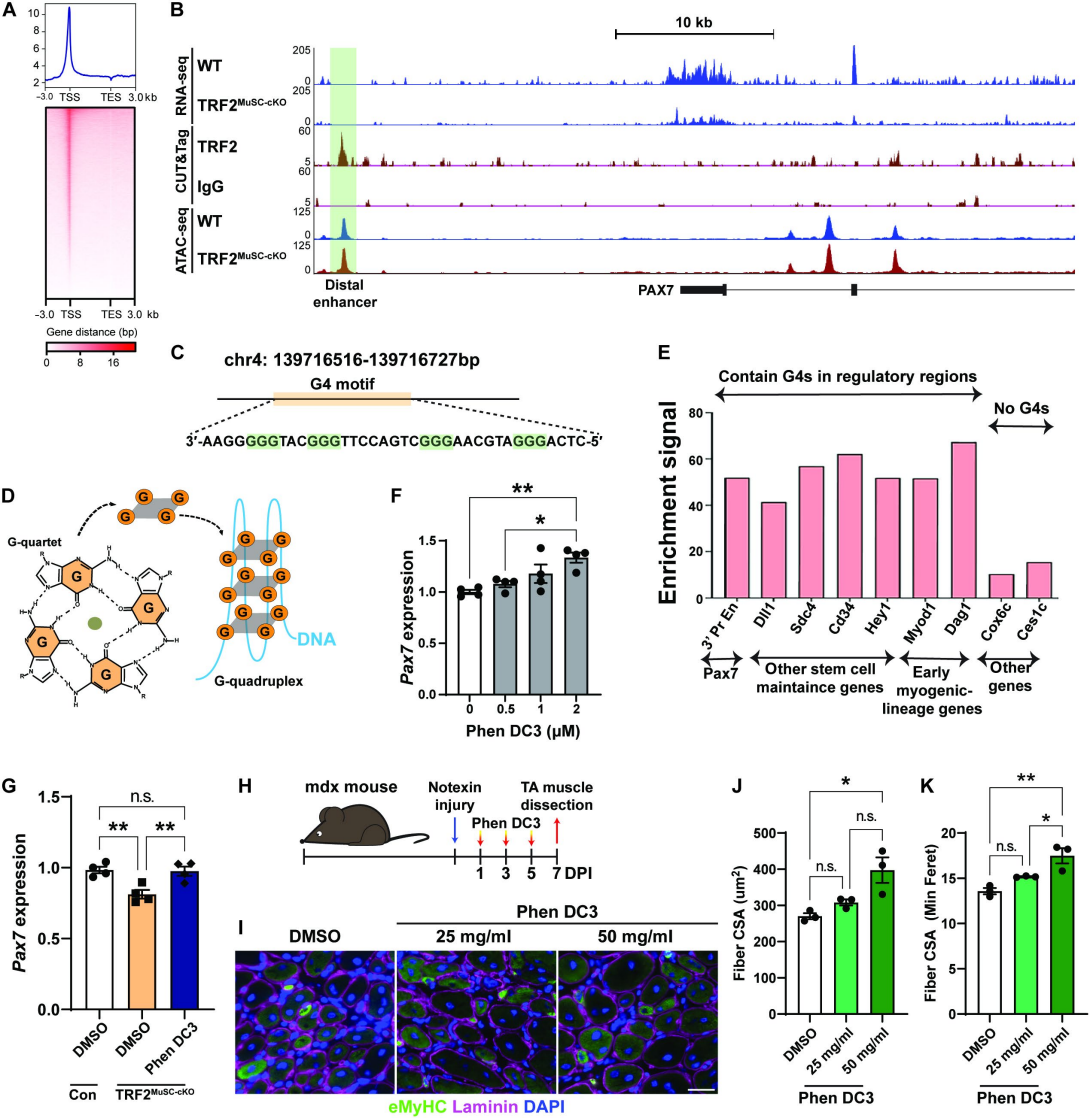
TRF2 maintains MuSC identity by directly regulating stem cell maintenance genes through G4-containing regulatory elements. (**A**) Quality control metrics for CUT&Tag chromatin profiling. bp, base pairs. (**B**) UCSC (University of California Santa Cruz) Genome Browser tracks showing TRF2 occupancy at the *Pax7* locus together with RNA-seq and ATAC-seq profiles from control and TRF2^MuSC-cKO^ MuSCs. The highlighted area in green denotes the TRF2-binding site identified by CUT&Tag. (**C**) DNA sequence of the TRF2-bound regulatory element within the *Pax7* locus containing G4 motifs. (**D**) Schematic of a G-quarter composed of four Hoogsteen–base-paired guanines that stack to form a DNA G4 structure. (**E**) TRF2 binding enrichment at the Pax7 locus and additional down-regulated stem cell maintenance genes. (**F**) qRT-PCR analysis of *Pax7* expression following treatment and increasing concentrations of PhenDC3, a specific G4-stabilizer compound (*n* = 4 per condition). (**G**) qRT-PCR analysis of *Pax7* expression in control and TRF2^MuSC-cKO^ MuSCs treated with PhenDC3 (2 μM) (*n* = 4 mice per genotype and condition). (**H**) Experimental design for PhenDC3 treatment in mdx mice following muscle injury. Mice received PhenDC3 at 1, 3, and 5 DPI, and muscle morphology was assessed at 7 DPI. (**I**) Representative images of regenerating fibers within the injured region. Newly forming myofibers stained for eMyHC, nuclei (DAPI), and myofiber boundaries (laminin) in muscles from mdx mice following treatment with vehicle [dimethyl sulfoxide (DMSO)] PhenDC3 (25 and 50 μg/ml). Scale bar, 20 μm. (**J**) Quantification of myofiber CSA normalized per micrometer tissue and (**K**) the minimum Feret diameter in vehicle-treated (white bars) and PhenDC3-treated (green bars) muscles (*n* = 3 mice per condition). Data are presented as means ± SEM. Statistical analysis was performed using one-way ANOVA with Tukey’s multiple comparisons test in [(F), (G), (J), and (K)]. *P < 0.05; **P < 0.01; n.s., not significant. TSS, transcription start site; TES, transcription end site.

It has been suggested that, in addition to its canonical role in telomere protection (*42*, *51*), TRF2 can also bind to nontelomeric DNA, thereby modulating chromatin architecture (*58*–*60*) and transcriptional activities of genes (*61*–*67*). To determine whether the permissive or instructive role of TRF2 in maintaining MuSC stemness is mediated through its nontelomeric function as a transcriptional regulator of stem cell gene(s), we examined genome-wide TRF2 binding in MuSCs using Cleavage Under Target and Tagmentation (CUT&Tag) (*68*, *69*). This population-level chromatin occupancy analysis identified that most of TRF2 binding was associated with telomeres and showed a strong preference for the TTAGGG telomere repeat sequence (fig. S14A), validating the specificity of detectable TRF2-binding sites. We note that CUT&Tag provides population-level chromatin occupancy information and is complementary to single-cell IF/PNA-FISH colocalization approaches. However, we also detected extensive nontelomeric binding of TRF2 at sites distributed across the genome (fig. S14A), consistent with previous reports indicating that TRF2 can occupy extra-telomeric sites (*70*, *71*). Notably, extra-telomeric TRF2 binding occurred predominantly at gene promoters, although substantial binding was also observed within intronic and distal intergenic regions (Fig. 7A and fig. S14B).

To define the nontelomeric, transcriptional regulatory role of TRF2 in MuSCs and corroborate the differential expression of stem cell genes in TRF2^MuSC-cKO^ MuSCs (Fig. 4A), via TRF2 binding at the nontelomeric cis-regulatory elements, we used an integrative approach combining genomic and transcriptomic analyses. This approach revealed that TRF2 is required for the expression of stem cell–associated genes in MuSCs as ∼45% of the down-regulated genes in TRF2^MuSC-cKO^ MuSCs harbor TRF2-binding sites, compared with only ∼19% of the up-regulated genes (fig. S15A). We next analyzed the DNA sequences underlying the 20,277 TRF2 peaks and observed that ∼50% of the peaks (10,413 peaks corresponding to 8395 genes) contained high-confidence G4-forming sequences, as independently identified by two distinct algorithms (gquad or pqsfinder). G4s are alternative DNA secondary structures that are self-assembled from guanine-rich sequences (*72*). Intriguingly, previous studies have demonstrated that TRF2 associates with DNA-G4s (*59*, *65*, *71*, *73*–*76*). In agreement with these findings, deeper analysis of the direct transcriptional targets of TRF2 in MuSCs, which were also down-regulated in TRF2^MuSC-cKO^ MuSCs (622 genes), revealed that most of the genes (89.7%) contained putative G4 sequences within TRF2-bound regulatory regions (fig. S15B). TRF2 peaks associated with predicted G4 motifs were predominantly found at gene promoters, although it was also present in intronic and distal intergenic regions (fig. S15C). To assess whether TRF2 loss alters chromatin accessibility, we performed assay for transposase-accessible chromatin with high-throughput sequencing (ATAC-seq) in wild-type and TRF2^MuSC-cKO^ MuSCs. Genome-wide comparison revealed that most accessible regions were shared between conditions (35,177 peaks), indicating that loss of TRF2 does not broadly disrupt global chromatin accessibility (fig. S16). We did not observe changes in chromatin accessibility at TRF2 target genes that change their expression upon TRF2 ablation (fig. S16). These findings support a model in which TRF2 contributes to transcriptional output at selected target genes without broadly altering chromatin accessibility at their regulatory elements.

Among the genes identified by these analyses, *Pax7* was particularly notable because of its central role in MuSC identity (*4*, *22*, *23*, *25*, *77*). CUT&Tag revealed TRF2 binding at the *Pax7* locus at an ENCODE-annotated enhancer (Fig. 7B) containing G4-forming sequences (Fig. 7, C and D). ATAC-seq analysis indicated that TRF2 is not required to maintain chromatin accessibility at this enhancer, despite marked changes in gene expression upon its ablation. These findings identify *Pax7* as a TRF2-associated transcriptional target whose expression changes despite preserved chromatin accessibility at the enhancer. *Pax7* was not unique in this respect: Comparable TRF2 enrichment was observed at regulatory regions of multiple down-regulated genes linked to stem cell maintenance (*Dll1*, *Sdc4*, *CD34*, and *Hey1*) and early myogenic-lineage progression (*MyoD1* and *Dag1*) (Fig. 7E). Together, these data support the idea that TRF2 is associated with a broader G4-enriched regulatory network that contributes to MuSC identity and myogenic progression, rather than acting through a single downstream locus.

Emerging evidence from cell lines suggests that G4s can serve as recruitment hubs for regulatory factors that orchestrate cell fate transitions (*59*, *63*, *65*, *71*). Intriguingly, G4s have also been recently shown to be prevalent in enhancers and promoters of lineage-specific transcription factors that maintain human ESCs pluripotency (*78*). We therefore used *Pax7* as a representative TRF2-responsive stem cell gene, given its central role in specifying the myogenic stem cell state (*4*). Because the integrative genomic analyses identified TRF2 occupancy together with G4-forming sequences at the *Pax7* regulatory region (Fig. 7, B to D), we next asked whether pharmacologic modulation of G4 structures would influence expression of TRF2-dependent program in MuSCs. To test this, wild-type MuSCs were treated with PhenDC3 (fig. S17B), a well-characterized modifier of G4 structures (*78*). Under these conditions, PhenDC3 caused a dose-dependent increase in *Pax7* expression (Fig. 7F). We next questioned whether G4 intervention could partially compensate for loss of TRF2. Treatment of TRF2^MuSC-cKO^ MuSCs with 2 μM PhenDC3 (fig. S17B) restored *Pax7* expression to levels comparable to those of control MuSCs (Fig. 7G). As PhenDC3 acts on DNA secondary structure, we considered the possibility that it might influence Cre-mediated recombination at the *Terf2* locus. However, *Terf2* expression was comparable between vehicle- and PhenDC3-treated TRF2^MuSC-cKO^ MuSC (fig. S17C), indicating that the transcriptional effect occurred independently of recombination efficacy. These findings suggest that pharmacologic G4 manipulation can partially restore a TRF2-dependent transcriptional program in MuSCs. To determine whether the effect of G4 modification extended beyond a single locus, we examined additional representative TRF2-bound, G4-motif containing genes, such as *Sdc4* and *MyoD1* (Fig. 7E). Similar to *Pax7*, both genes were down-regulated in TRF2^MuSC-cKO^ cells and were significantly restored by PhenDC3 treatment (fig. S17D). These observations support a broader functional influence of G4 modulation on multiple TRF2-associated loci. Consistent with this broader transcriptional effect, PhenDC3 also attenuated the impaired myogenic commitment of TRF2^MuSC-cKO^ cells (fig. S17E-G). Together, these data support a model in which targeting G4s functionally buffers a broader TRF2-dependent gene network relevant to myogenic lineage progression.

Last, to determine whether modulation of G4 biology could improve regenerative output in vivo, we used the *mdx* mouse model of DMD. Because *mdx* muscles undergo spatially asynchronous regeneration (*79*), we first induced a synchronized localized injury using notexin. PhenDC3 was then administered intramuscularly at 1, 3, and 5 DPI, and muscle regeneration was assessed at 7 DPI (Fig. 7H). Vehicle-treated *mdx* muscles served as controls, allowing direct assessment of PhenDC3 effects in a dystrophic context. PhenDC3 treatment significantly increased the size of newly forming eMyHC^+^ fibers in *mdx* mice compared with those in vehicle-treated controls (Fig. 7, I to K). This experiment provides an in vivo functional evidence that modulation of G4-associated regulation can improve myogenic outcome in a dystrophic setting. Collectively, these results indicate that extra-telomeric TRF2 associates with regulatory regions enriched for predicted G4-forming sequences at pivotal stem cell and early myogenic genes and that modulation of G4 stability functionally influences this transcriptional program and myogenic output.

## DISCUSSION

Binding of TRF2 to telomeric DNA protects mammalian chromosome ends from being recognized as DNA breaks (*80*) and from undergoing end-to-end fusions (*42*, *52*). By suppressing DDR activation at telomeres, TRF2 preserves telomere stability and prevents pathological outcomes associated with telomere dysfunction, including cancer and age-related diseases (*81*). Although the telomere-protective machinery and the role of TRF2 have been studied extensively in cancer cells (*61*, *80*, *82*–*87*) and in a limited number of mammalian tissues (*8*, *19*, *64*, *88*–*90*), its role in muscle injury and regeneration has remained poorly understood. Here, we uncover a mechanism by which TRF2 preserves the myogenic identity of MuSCs through a previously unrecognized extra-telomeric function. Specifically, we discovered that TRF2 occupies regulatory regions of lineage-defining stem cell and early myogenic genes, within a broader G4-enriched transcriptional network that supports the myogenic program. Following injury, TRF2 levels change dynamically as MuSCs exit quiescence and enter the myogenic lineage and later return toward a quiescent state. The upstream injury-responsive pathways that regulate *Terf2* expression remain to be defined, and coordinated regulation of *Terf2* and other MuSC identity genes by broader injury-associated signaling pathways cannot be excluded. Yet, in the absence of TRF2, MuSCs fail to maintain stemness, leading to defective stem cell function and severe regeneration defects after injury (fig. S18).

MuSC-mediated regeneration after acute injury is a highly coordinated process that is essential for preserving tissue integrity after muscle damage caused by trauma or overuse (*91*, *92*). Muscle injury activates quiescent MuSCs, triggering their expansion and entry into the myogenic program. This program governs the balance between proliferation and differentiation and drives the transition from proliferating myoblasts to a syncytial contractile myofibers (*91*). It is well established that the balance between quiescence and proliferation in stem cell populations is tightly regulated as its disruption can lead to excessive cell growth, impaired tissue homeostasis, or defective repair (*1*). However, the exact molecular signatures guiding the shift from quiescence to reparative myogenesis remain incompletely defined (*93*).

Emerging evidence from cancer biology suggests that G4s can function as recruitment hubs for regulatory factors, shape cell-type–specific transcriptomes, and modulate chromatin accessibility during cell fate transitions (*59*, *63*, *65*, *71*). These observations raise the possibility that G4s may represent an additional layer of epigenetic regulation of the transcriptional machinery. Although TRF2 has been previously reported to bind extra-telomeric G4s in cell lines (*59*, *65*, *71*, *73*–*76*), the role of TRF2-G4 interactions in context-dependent transcriptional control has not been established in vivo. Recent work in human ESCs showed that G4s are linked to stem cell pluripotency and that G4 maintenance helps stabilize transcription of genes required for stem cell plasticity (*78*). Yet, how G4s and their associated regulatory factors coordinate tissue-specific stem cell programs remains an unexplored and exciting research area.

Our findings place TRF2 within this emerging framework. We show that TRF2 is dynamically regulated across distinct MuSC states following injury. Genome-wide analysis revealed that extra-telomeric TRF2 peaks are enriched at proximal promoter regions, with additional localization at intronic and distal intergenic elements. Integration of CUT&Tag and RNA-seq datasets further showed that TRF2 occupancy is preferentially associated with genes that are down-regulated upon TRF2 deletion and that these TRF2-bound regulatory regions are highly enriched for G4-forming motifs. Together, these results support a model in which TRF2-G4 interactions contribute to the transcriptional regulation of stem cell genes in MuSCs. Our data are most consistent with a pathway-level role for TRF2 in supporting a G4-dependent transcriptional network, rather than with a model requiring exhaustive biochemical dissection of any single target locus. These data place TRF2 within a broader G4-associated regulatory program that is required to preserve MuSC identity and regenerative competence. In these settings, TRF2 does not act as a classical pioneer factor. The preservation of chromatin accessibility at TRF2-associated target loci, including the *Pax7* enhancer, suggests that TRF2 does not primarily regulate these genes through large-scale chromatin opening. Instead, TRF2 may influence transcriptional output through mechanisms operating downstream or independently of accessibility. Potential mechanisms include recruitment or stabilization of transcriptional coregulators, modulation of enhancer-promoter communication, effects on local DNA topology or higher-order chromatin architecture, regulation of R-loop–associated structures, or control of RNA polymerase II pause-release dynamics. Consistent with this possibility, recent studies have implicated G4-containing regulatory regions in RNA polymerase II pause-release and transcriptional elongation (*94*). Defining which of these mechanisms operates at individual MuSC loci will require dedicated biochemical and structural studies. Regardless of the exact mechanism, TRF2 sustains MuSC quiescence and lineage identity not by preventing cell death or senescence, but by stabilizing the transcriptional state required for stem cell function. Consistent with this view, lineage-traced TRF2-deficient cells persist yet no longer fulfill canonical MuSC criteria.

The findings reported here also broaden the biological significance of extra-telomeric TRF2 activity beyond the settings in which it has previously been described. TRF2 has been implicated in regeneration in liver and lung tissues (*8*, *88*). Liver regeneration was not affected by TRF2 depletion (*88*), whereas lung regeneration was severely impaired after alveolar stem cell–specific TRF2 removal (*8*). In addition, epithelial-specific TRF2 ablation disrupts skin homeostasis (*19*). Although the consequences of TRF2 loss in vivo vary across tissues, DDR activation is a common feature of these TRF2-deficient settings (*8*, *19*, *88*). By contrast, our findings indicate that the regenerative failure observed in TRF2^MuSC-cKO^ muscle is not explained by the canonical telomeric role of TRF2. In MuSCs lacking TRF2, we detected no telomeric or global DDR activation under the conditions assayed, no differences in telomere length, no evidence of widespread telomere fusion-associated dysfunction, no increase in apoptosis or senescence, no dependence on the TRF2-p53 axis, and no defect in proliferation. These observations distinguish MuSCs from tissues in which TRF2 loss primarily causes pathology through telomere deprotection and DDR engagement.

This distinction may be especially relevant in skeletal muscle, a tissue with high regenerative capacity but low carcinogenic potential (*95*, *96*). Although skeletal muscle constitutes a large fraction of the human body mass, primary cancers and metastases are rare in muscle (*95*, *96*), with rhabdomyosarcoma being an uncommon exception (*97*). Likewise, cancer cachexia reflects systemic tumor-driven wasting rather than malignant transformation of muscle itself (*98*). It is therefore plausible that skeletal muscle handles pathways linked to telomere protection, cell fate, and tumor suppression differently from highly proliferative tissues. MuSCs are long-lived stem/progenitor cells with strong self-renewal capacity, resilience, and strict unipotency as they are committed exclusively to the muscle lineage (*99*). Our findings that TRF2 is dynamically regulated across MuSCs states after injury, to our knowledge not previously described in adult resident stem cells, suggest that modulation of TRF2 levels may be part of a tissue-adapted strategy that supports regeneration while minimizing oncogenic risk. Although this study is not centered on cancer biology, it reveals a mechanism by which MuSCs preserve stemness independently of overt telomere dysfunction.

We found that, besides an absence of telomeric or global DDR activation in TRF2^MuSC-cKO^ MuSCs, there are also no differences in telomere length, apoptosis, senescence, or dependency on the TRF2-p53 axis, whereas MuSCs with TRF2-deletion exhibited normal proliferation rates. The results presented here further highlight that muscle regeneration failure of TRF2^MuSC-cKO^ muscle is not a consequence of conventional telomeric TRF2 function reported for cells or tissues prone to cancer.

How MuSCs maintain telomere protection in the absence of TRF2 remains an important open question. Prior work showed that TRF2 knockdown in differentiated myotubes does not trigger a telomeric DDR (*64*). Instead, telomere protection in those cells is supported by a noncanonical function of FOXO3, which becomes up-regulated and binds TRF2-deficient telomeres to protect them from ATM-dependent DDR activation (*90*). In contrast to these fully differentiated muscle cells, our studies demonstrated that TRF2 ablation does not trigger DNA damage at telomeres in MuSCs. In somatic cells more broadly, TRF2 suppresses recognition of telomeres as DNA double-strand breaks by ATM and 53BP1, thereby preserving genomic integrity (*18*). During DNA replication, telomeres transiently shift from a closed to a more open configuration, exposing the 3′ overhang and potentially rendering chromosome ends vulnerable to DDR machinery and end-to-end fusion (*100*, *101*). In this context, TRF2 suppresses ATM autophosphorylation and downstream activation of factors such as γH2AX, 53BP1, and p53, and its loss commonly gives rise to telomere dysfunction-induced foci. However, this paradigm is not universal. In embryonic stem cells, TRF2 loss was reported to be compatible with viability and proliferation (*102*, *103*), suggesting that TRF2 is not absolutely required for telomere protection in all cell types. Our findings extend this concept to adult MuSCs and raise the possibility that MuSCs, like ESCs, have an alternative mechanism of telomere protection. Whether this mechanism resembles that described in ESCs or instead reflects a distinct adaptation of adult tissue stem cells remains to be determined.

The work presented here goes beyond showing that TRF2 is dispensable for telomere protection in a specific cell type. We demonstrate that a noncanonical, extra-telomeric function of TRF2 is linked to tissue regeneration, contributes to dystrophic pathology, and is functionally responsive to G4 stabilization. In this way, our study identifies an early stem cell–protective mechanism operating during chronic muscle injury and suggests that maintenance of G4-dependent regulatory programs may help preserve myogenic competence. More broadly, these findings provide a framework for identifying additional molecules that cooperate with TRF2 as MuSCs transition between quiescence, activation, differentiation, and self-renewal. They also open the door to dissecting how telomeric and nontelomeric TRF2 functions contribute differently to MuSC dysfunction at distinct stages of muscle disease and whether similar principles operate in other adult stem cell compartments. In summary, this study identifies a previously unrecognized noncanonical telomeric function of TRF2 in adult resident stem cells from a tissue with high regenerative capacity and low carcinogenic potential.

## MATERIALS AND METHODS

### Animals

All animals were maintained and bred following the Institutional Animal Care and Use Committee guideline of University of Pennsylvania. C57BL/6 (JAX stock no. 000664), B6-DMD^mdx4cv^ (JAX stock no. 002378) (*104*), Pax7^creER (Gaka)^ (JAX stock no. 017763) (*105*), p53^LoxP^ (JAX stock no. 008462) (*106*), R26^H2B-mcherry^ (JAX stock no. 023139) (*107*), R26^tdTomato^ (JAX stock no. 007909) (*108*) were obtained from the Jackson Laboratory. The Pax7EGFP mice used in this study are described in (*14*). TRF2cKO MEFs and TRF2^Loxp^ mice are described in (*88*). All experimental mice were maintained on a C57BL/6 background. For two-photon microscopy, Pax7EGFP mice were crossed with the designated mouse lines, as described in the manuscript. To induce Cre expression, 3 mg of Tam per 20 g of body weight (dissolved in 10% ethanol in corn oil) was administrated by intraperitoneal injection every other day for a total of seven administrations. Tam injection was started at 8 to 10 weeks of age except for dystrophic mdx^4cv^ mice that received Tam at 6 weeks of age. Mice less than a week age difference were used for experimental analysis. Both males and females were used in this study using wild-type mice, but gender-matched cohorts were compared for all experiments, except the ones involved dystrophic (mdx) mice. Because the DMD primarily appears in boys, only males were used for mdx mice experiments. To dissect muscles, all mice in this study were euthanized by CO_2_ asphyxiation, followed by cervical dislocation.

### Muscle injury

Mice were anesthetized using 1 to 1.5% isoflurane in oxygen (1.5 ml/min) and subjected to muscle injury. TA muscles were injected with 10 μl of notexin (10 μg/ml, Latoxan), as we previously described (*14*, *17*). Postinjured muscles were dissected at designated time point.

### MuSC isolation

MuSCs were isolated using fluorescence-activated cell sorting (FACS), as previously described (*14*, *46*, *109*, *110*). More specifically, mice were euthanized, and all hindlimb muscles were dissected. Collected muscles were minced and placed in a C tube (Miltenyi) containing 0.15% collagenase (Sigma-Aldrich) in Dulbecco’s modified Eagle’s medium (DMEM). Tubes were loaded into magnetic-activated cell sorting (MACS) dissociator (Miltenyi) and subjected to manufacturer’s muscle-01 program. Tubes were incubated at 37°C for 30 min, followed by the muscle-01 program again, and incubated additional 10 min at 37°C. Seventy-five microliters of 2% collagenase and same volume of dispase (4.8 U/ml; Roche) were added into tubes and incubated 30 min at 37°C. Muscle slurry was further dissociated using 21-gauge needle syringe and filtered through a 40-μm cell strainer. Filtered cells were collected 50-ml conical tubes containing 10 ml of DMEM:F12 medium supplemented with 10% fetal bovine serum (FBS). Cells were centrifuged at 300*g*/4°C and incubated in 1 ml of red cell lysis buffer (Thermo Fisher Scientific) for 5 min at room temperature. For linage-negative selection, cells were suspended in FACS buffer [2.5% goat serum and 2 mM EDTA in phosphate-buffered saline (PBS)] containing biotin-conjugated antibodies targeting CD45, CD31, CD11b, and Sca1 and incubated for 1 hour on ice. Cells were pelleted and resuspended in FACS buffer containing antibodies targeting VCAM (or CD34) and α7-integrin, as well as streptavidin conjugated chycoerythrin-cyanine 7 (PE-Cy7). Cells were incubated for 1 hour in dark on ice and resuspended in FACS buffer containing viability dye 7-AAD (finical concentration, 4 μg/ml). The CD45^−^/CD31^−^/CD11b^−^/Sca1^−^ and VCAM^+^ (or CD34^+^)/α7-integrin^+^ cells were used to identify MuSCs.

### Isolated MuSC culture

FACS-isolated MuSC resuspended myoblast growth medium [myoblast GM, Ham’s F10 medium supplemented with 20% FBS, 1× anti-anti, and basic fibroblast growth factor (2.5 ng/m)] and plated on poly-l-lysine (MilliporeSigma)/laminin (MilliporeSigma)–coated chamber slide (Thermo Fisher Scientific). MuSCs were attached for 1 hour at 37°C incubator with 5% CO_2_. Attached MuSCs were fixed in 4% paraformaldehyde in PBS before staining or cultured in myoblast GM for further experiments. The in vitro TRF2 deletion was induced by culturing isolated MuSCs in 4-OHT containing medium for indicated time. Designated concentrations of Phen-DC3 (Sigma-Aldrich) were treated to 1 hour attached MuSCs for 48 hours. For myotube formation, cells were cultured in differentiation medium [DMEM supplemented with 2% horse serum, insulin (1 μg/ml), and 1× anti-anti] for designated time. Cells were fixed in 4% paraformaldehyde in PBS for immunofluorescence staining.

### MEF culture

TRF2 F/− Rosa26-CreERT2 cells were gifted by E. Lazzerini-Denchi. MEFs were cultured in DMEM supplemented with 10% FBS and 1% anti-anti. Cells were maintained in 5% CO_2_ incubator at 37°C. For experiment, 3000 MEFs cells were plated in chamber slide (Thermo Fisher Scientific) and treated with 1 μM 4-OHT or ethanol containing medium for designated time. Cells were fixed in 4% paraformaldehyde for 15 min at room temperature and subjected to IF or TIF staining.

### TUNEL apoptosis assay

Degree of cellular apoptosis was accessed by using TUNEL apoptosis assay that labels DNA strand breaks, following the manufacturer’s guidelines (Thermo Fisher Scientific). The FISCs were plated on laminin-coated (Sigma-Aldrich) chamber slides for 1 hour and fixed in 4% paraformaldehyde for 15 min and subjected to TUNEL staining. For in vitro short time apoptosis assay, FACS-isolated MuSCs from mice without Tam administration were plated on laminin-coated chamber slides. MuSCs were allowed to attach for 1 hour, and Cre-activation was induced by culturing them in myoblast GM containing 1 μM (4-OHT) for 72 hours. Cre-activated MuSCs were subjected to RNA isolation or fixed for TUNEL staining. Cells treated with 1 unit of deoxyribonuclease (Invitrogen) and 3 μM Staurosporine (Thermo Fisher Scientific) for 3 hours were used as technical and biological positive controls, respectively. At least 100 MuSCs per sample were analyzed to determine the percentage of TUNEL-positive MuSCs.

### Muscle tissue dissection and histological analysis

Diaphragm and hindlimb muscles (quadriceps, gastrocnemius, and TA muscles) were dissected and snap frozen in liquid nitrogen chilled 2-methlybutane (Sigma-Aldrich). Ten-micromer-thickness muscle sections were prepared by using cryostat (Leica, CM1950) Hematoxylin and eosin (Thermo Fisher Scientific) or trichrome (Sigma-Aldrich) staining were performed, following the manufacturer’s protocol. Images were taken by using Nikon Ni wide-field epifluorescence microscope. Muscle fiber cross sectional areas and fibrotic areas were manually measured by using ImageJ/Fiji.

### RNA isolation and qRT-PCR

RNA of FACS-sorted MuSCs or cultured MuSC was isolated using an RNeasy Plus micro kit (QIAGEN), according to the manufacturer’s instructions. cDNA was generated with the Protoscript II First Strand cDNA synthesis kit (New England Biolabs). qRT-PCR was conducted on a Quantstudio 6 instrument (Applied Biosystems) using TaqMan primers (Applied Biosystems). Primers used in this study were listed in table S1. Gene expression was determined using ΔΔCt method.

### Immunofluorescent staining

One-hour attached MuSCs were fixed in 4% paraformaldehyde in PBS for 15 min and permeabilized in 0.5% Triton X-100 in PBS for 10 min. MuSCs were blocked with blocking solution [3% bovine serum albumin (BSA; w/v) and 0.1% Triton X-100 in PBS] for 1 hour. Primary antibodies diluted in blocking solution were applied and incubated overnight at 4°C. Primary antibodies include mouse Pax7 [1:10, Developmental Studies Hybridoma Bank (DSHB)], rabbit TRF2 (1:100, Novus Biologicals), rabbit γH2AX [1:500, Cell Signaling Technology (CST)], and rabbit 53BP1 (1:1000, Novus Biologicals). Secondary antibodies were added to blocking solution and incubated for 1 hour at room temperature in dark. Cells were washed in PBS and mounted with ProLong Gold with 4′,6-diamidino-2-phenylindole (DAPI; Thermo Fisher Scientific). Fluorescence intensities were measured by using ImageJ/Fiji. Muscle sections were fixed in 4% paraformaldehyde in PBS for 10 min and permeabilized in 0.5% Triton X-100 in PBS for 10 min. Tissues were blocked in tissue blocking solution (5% goat serum, 2% BSA, and 0.1% Triton X-100 in PBS) for an hour at room temperature. Primary antibodies were made in tissue blocking solution and incubated overnight at 4°C. The primary antibody titrations used in this study are Pax7 (1:10, DSHB), TRF2 (1:50 Novus Biologicals), perilipin (1:100, CST), laminin (1:200, Thermo Fisher Scientific), eMyHC (1:5, DSHB), and wheat germ agglutinin (1:400, Thermo Fisher Scientific). Secondary antibodies diluted in tissue blocking solution were applied for an hour at room temperature followed by mounting with ProLong Gold with DAPI (Thermo Fisher Scientific). Goat anti-mouse, goat anti-rabbit, and goat anti-rat secondary antibodies were diluted in 1:1000, 1:500, and 1:1000 ratio, respectively, in both cell and tissue immunostaining. Antibodies used in this study were listed in table S2.

### SA-β-gal staining

Senescence-associated beta-galactosidase (SA-β-gal) activity was determined in FISCs or myoblasts according to the manufacturer’s instruction (CST). FISCs were allowed to attach on laminin-coated chamber slides for 1 hour and fixed for SA-β-gal staining. Myoblast were treated with bleomycin (1 μg/ml) for 48 hours to induce cellular senescence and fixed for SA-β-gal staining. More than 100 cells were quantified to enumerate SA-β-gal–positive cells.

### EdU proliferation assay

For in vivo MuSC proliferation assay, EdU solution (100 mg/kg) in PBS was injected intraperitoneally into 1-day postinjured mice. One day after EdU administration, mice were euthanized, and injured TA muscle was collected. TA muscles were digested into single cells following the protocol described in MuSC isolation. Cells were suspended in FACS buffer (2.5% goat serum and 2 mM EDTA in PBS) containing biotin-conjugated antibodies targeting CD45, CD31, CD11b, and Sca1 and incubated for 1 hour on ice. Cells were pelleted and resuspended in FACS buffer containing antibodies targeting VCAM and α7-integrin, as well as streptavidin conjugated PE-Cy7. Cells were incubated for 1 hour in dark on ice and resuspended in FACS buffer and subsequently subjected to EdU assay using the Click-iT EdU Alexa Fluor 488 Flow Cytometry Assay Kit according to the manufacturer’s instructions. The percentage of EdU-positive MuSCs within CD45^−^/CD31^−^/CD11b^−^/Sca1^−^ and VCAM+/α7-integrin+ MuSCs was determined by flow cytometry analysis.

### Whole–TA muscle imaging using two-photon microscopy

To enumerate the MuSC in their native environment, TA muscle dissected from control (Pax7EGFP) and Pax7EGFP-TRF2^MuSC-cKO^ mice were imaged using two photons, as described in (*17*, *111*). Briefly, dissected TA muscles were fixed in 4% paraformaldehyde for 2 hours at 4°C in dark and gently washed in PBS. Muscles were mounted on a customized chamber slide. High-resolution serial sections were imaged from the middle of the TA muscle using a Leica SP* Confocal/Multiphoton Microscope system. Serial optical sections were collected every 1.5 μm, flattened, and normalized to volume scanned. Quantification and morphological analysis of EGFP-positive MuSCs were conducted by using ImageJ/Fiji.

### MicroCT imaging

In vivo whole-mouse scanning was performed using a preclinical microCT imaging system (vivaCT 40, Scanco Medical AG, Brüttisellen, Switzerland) at 38-μm nominal voxel size. Mice were anesthetized using 1 to 1.5% isoflurane in oxygen (1 to 1.5 ml/min) and immobilized in a customized holder. The scanner was operated at 55-kVp energy and 145-μA intensity. Images were generated with 200-ms integration time and one signal average. Kyphotic index was measured as previously described (*112*).

### EBD uptake analysis

EBD (10 ml/kg) in PBS (10 mg/ml) was injected into mice by intraperitoneal injection. Diaphragm and gastrocnemius muscles were dissected after 18 to 20 hours post–EBD injection. Muscles are snap frozen in liquid nitrogen chilled 2-mythlybutane and sectioned in 10-μm thickness. Whole–muscle section images were obtained using Axio Scan.Z1 slide scanner (Zeiss, USA).

### Telomere length analysis of FACS-isolated MuSCs

MuSC telomere length was measured using quantitative FISH (Q-FISH) of Cy3-conjugated telomere probe (TelC-Cy3, PNR Bio), as previously described (*45*). FACS-isolated MuSCs were plated on poly-l-lysine/laminin-coated chamber slide and allowed to attach for 1 hour. MuSCs were fixed in 4% paraformaldehyde and permeabilized with 0.1% Tween 20 in PBS. Ribonuclease A (100 μg/ml; Thermo Fisher Scientific) were applied for 20 min at 37°C, followed by incubation in preheated TelC probe (1:300 dilution of 50 μM TelC probe) in Q-FISH buffer [60% formamide, 5% 10× nucleic acid hybridization blocking buffer, and 2% 1 M tris (pH 7.5) in deionized water] for 6 min at 86°C. Cells were cooled 4 hours at room temperature in dark. Slides were washed twice in pre warmed (at 56°C) 2×, 1×, and 0.5× saline sodium citrate buffer for 10 min each. Cells were rinsed with PBS and mounted with ProLong Gold with DAPI (Thermo Fisher Scientific). Images were acquired using a Nikon eclipse 90i wide-field epifluorescence microscope. Seven to 10 *z*-stacks were taken for both telomere (Cy3) and DAPI at 1-μm step. Telomere length was analyzed using telomere. At least 100 cells per biological replicate were measured for comparison.

### Telomeric DNA damage (TIF) analysis

Telomeric DNA damages in MuSCs were determined as previously described (*109*). Briefly, MuSCs were isolated without Tam administration and plated on laminin-coated chamber slides for 1 hour. Attached MuSCs were cultured in 1 μM 4-OHT containing myoblast GM for 60 hours and fixed in 4% paraformaldehyde for 15 min. Fixed cells were subjected to telomere staining following the procedure described in the “Telomere length analysis of FACS-isolated MuSCs” section. PBS rinsed cells were incubated in blocking solution (3% BSA and 0.1% Tween 20 in PBS) for 1 hour at room temperature. 53BP1 antibody (1:1000, Novus Biologicals) diluted in blocking solution was applied for overnight at 4°C. Secondary antibody was treated for 1 hour at room temperature and followed by mounting with ProLong Gold with DAPI (Thermo Fisher Scientific). Images were acquired using a Nikon eclipse 90i wide-field epifluorescence microscope. Seven to 10 *z*-stacks were taken for telomeres, DNA damages (53BP1), and DAPI at every 1-μm step. At least 100 cells were analyzed per biological replicates to determine the degree of telomeric DNA damages.

### RNA isolation, library preparation, and sequencing

RNA extraction and purification followed the manufacturer’s protocol from Direct-zol RNA MiniPrep (Zymo Research Corporation). Final RNA concentrations were measured using a Qubit Fluorometric Quantification Instrument (Thermo Fisher Scientific). RNA-seq libraries were generated according to the manufacturer’s instructions, CORALL Total RNA-Seq Library Prep from Lexogen GmbH (www.lexogen.com) was used as it supports a wide RNA quality and input range. Unique Dual Indexes Primer Pair Set was incorporated for multiplexed high-throughput sequencing. The final product was assessed for its size distribution and concentration using the Bioanalyzer High Sensitivity DNA Kit (Agilent Technologies). Shortly, 1 ng of input RNA was first depleted of ribosomal RNA, and then library generation was initiated by random hybridization of Displacement Stop Primers to the RNA template. These primers contain partial Illumina-compatible P7 sequences. Reverse transcription extends each DSP to the next DSP where transcription is effectively stopped. This stop prevents spurious second strand synthesis and thus maintains excellent strand specificity and seamlessly integrates unique molecular identifiers. UDI indexes were added during the PCR amplification step, in which complete adapter sequences required for cluster generation on Illumina instruments were also added. All purification steps were based on magnetic beads (Beckman Coulter). The resulting libraries were pooled, diluted to 2 nM using 10 mM tris-HCl (pH 8.5), denatured, and loaded onto an P2-100 (PE50) flow cell on an Illumina NextSeq 1000 (Illumina Inc.) as per the manufacturer’s instructions. Demultiplexed and adapter-trimmed sequencing reads were generated using Illumina bcl2fastq.

### RNA-seq data analysis

Corall adapter sequences were integrated into the TrimGalore module. Quality control was carried out using FastQC v0.11.9. High-quality reads were then aligned to the Ensembl GRCm38 reference genome index using both STAR v2.7.10a and Salmon for alignment and quantification. The following software versions were also used in the pipeline: Samtools v1.15.1, RSeQC v3.0.1, Qualimap v2.2.2-dev, and Preseq v3.1.1. The count files generated from salmon were analyzed in R (version 4.3.0) in R studio (version 3.1.446). The R package edgeR (version 3.42.4) was used to process the raw counts by filtering, library scaling, and converting to a log counts per million with a trimmed mean of the *M*-values normalization. The principal components analysis plot was generated using the R package graphics (version 4.3.0) using the plot function. The limma package (version 3.56.2) was used for differential expression analysis using a robust method for fitting the linear model and for computing the empirical Bayes statistics. Differentially expressed genes with an adjusted *P* value less than 0.02 were considered significant. The volcano and scatter plots were generated using the R package ggplot2 (version 3.4.3). Heatmaps were generated using the R package pheatmap (version 1.0.12). The enriched terms for the bar plot were generated using the KEGG_2021_Human Gene-set Library (terms, 320; gene coverage, 8078; genes per term, 102) from EnrichR. Down-regulated genes in TRF2^MuSC-cKO^ compared with those in control with adjusted *P* value less than 0.02 were used to feed into the database. Plotted are the top enriched pathways with an adjusted *P* value less than 0.02. The gene-concept network diagrams were generated using the clusterProfiler package (version 4.8.3), msigdbr package (version 7.5.1), and the enrichplot package (version 1.20.1). Enriched terms were generated using the enricher function from the clusterProfiler package for any differentially expressed genes of TRF2^MuSC-CKO^ MuSCs relative to control with an adjusted *P* value less than 0.05. The TERM2GENE parameter was specified using the msigdbr database with species set to “*Mus musculus*,” category set to “C5,” subcategory set to “GO:BP,” and a “Benjamini-Hochberg” adjusted *P* value correction was applied to the enricher function. The top biological processes with an adjusted *P* value less than 0.05 were plotted using the cnetplots form the enrichplot package. The RNA-seq data have been deposited in the NCBI Gene Expression Omnibus and are accessible through GEO Series accession number GSE263262.

### CUT&Tag sequencing

To identify the TRF2-binding sites across the genome, CUT&Tag studies were done in MuSCs (45,000 cells) isolated as described in (*113*) from control (Pax7gCre/gCre;TdTomatofl/fl mice) TA and gastrocnemius muscles at 48 hours postinjury. MuSCs were immobilized on concanavalin A–coated magnetic beads and permeabilized with digitonin and incubated overnight with TRF2 antibody (Novus Biologicals, NB110-57130) at a dilution of 1:60. Upon tagmentation, libraries were prepared, as described in (*68*).

### ATAC-seq experiment

To assess chromatin accessibility genome-wide, ATAC-seq was performed on MuSCs from control or TRF2^MuSC-cKO^ mice. A total of 100,000 cells per condition were used for each experiment. Cells were pelleted and lysed to isolate nuclei, followed by incubation with Tn5 transposase (Active Motif) to simultaneously fragment DNA and insert sequencing adapters at accessible chromatin regions. Following tagmentation, libraries were amplified and purified, as described in (*68*).

### CUT&Tag and ATAC-seq analysis

High-throughput sequencing of CUT&Tag samples was processed first by trimming the adapters from raw reads with Cutadapt (v4.4). The trimmed reads were aligned to the mouse genome (mm10) with Bowtie2 (v2.5.1) using the following parameters “-local --very-sensitive-local --no-unal --no-mixed --no-discordant --phred33 -I 10 -X 700.” SAMtools (v1.10) and Picard (v3.1.1) (Broad Institute, 2019) were used for post–alignment processing, i.e., duplicates were marked and removed; nonuniquely aligned reads were filtered, and only reads mapped in proper pairs were retained for analysis. Normalized genomic coverage was calculated using bamCoverage (deepTools v3.5.3). The bamCoverage was used to generate bigWig tracks for visualization as well as normalized bedGraphs for downstream peak-calling. SEACR (v1.3) (*114*) was used to call “stringent” peaks for each bedgraph target file. MACS (v3.0.0) was also used to call peaks from the filtered alignments. Additionally, GoPeaks (v1.0.0) was used to call peaks. These peak sets were input to DiffBind (v3.10.1), whereby ENCODE blacklist regions were first removed before building a consensus peak set from the three peak callers. These peak regions were separated into unique regions for each sample and shared regions between samples. Nearest-gene annotations were obtained with ChIPseeker v1.36.0 from the consensus peak regions. Functional annotations of the resulting gene lists were compiled for GO and Kyoto Encyclopedia of Genes and Genome (KEGG) enrichment analysis using clusterProfiler (v4.8.3). Signal intensity heatmaps for the CUT&Tag libraries were generated using deepTools (v3.5.3). Using both normalized bigwigs and the M23 mouse gene annotation file from Gencode, ComputeMatrix was used with the scale-regions mode (--beforeRegionStartLength 3000, --regionBodyLength 5000, --afterRegionStartLength 3000). PlotHeatmap was used with the resulting matrix to plot the signal distribution. TelomereHunter (v1.1.0) was used to obtain summary statistics of telomere content using merged replicate alignments and immunoglobulin G samples as control with -mqt 15 and the banding file -b mm10cytoBand.txt from the UCSC genome database. The CUT&Tag-seq data have been deposited in the NCBI Gene Expression Omnibus and are accessible through GEO Series accession number GSE264045.

### Quantification and statistical analysis

All animal experiments were randomized. Data analysis and quantification was assessed by three independent investigators with blinded manner. At least three biological replicates were implemented in all the experiments. Specific sample size of each experiment is designated in the corresponding figure legends. Data processing and statistical analysis were conducted using GraphPad Prism 10 software. Experimental values are represented as means ± SEM. For comparison of two groups, two-tailed unpaired *t* test analysis with Welch’s correction was used. One-way analysis of variance (ANOVA) or two-way ANOVA test with Tukey’s correction was used for experiments having more than three groups as indicated in the figure legends. *P* value less than 0.05 was defined as statistically significant and significance levels were depicted as *P < 0.05, **P < 0.01, ***P < 0.001, and ****P < 0.0001.
