# Supplementary material for: TRF2 couples muscle stem cell identity to regenerative repair
Source: Sci Adv. 2026 Jul 31;12(31):eaei7316. doi: 10.1126/sciadv.aei7316 (PMC13426418; doi:10.1126/sciadv.aei7316)
Supplement: Supplementary file 1 — Supplementary Text Figs. S1 to S18 Tables S1 and S2 [file sciadv.aei7316_sm.pdf]

Science Advances
AAAS

Supplementary Materials for

TRF2 couples muscle stem cell identity to regenerative repair

Ji-Hyung Lee *et al.*

Corresponding author: Foteini Mourkioti, fmour@pennmedicine.upenn.edu

Sci. Adv. **12**, eaei7316 (2026)
DOI: 10.1126/sciadv.aei7316

This PDF file includes:

Supplementary Text
Figs. S1 to S18
Tables S1 and S2

Supplementary Text

MuSC-specific TRF2 deletion affects transition to myogenic commitment.

To evaluate the effect of TRF2 on early commitment towards myogenic differentiation, we first isolated cells from uninduced animals, treated them with 4-OHT for 72h (fig. S3A) and after evaluated *Terf2* deletion (fig. S3B), we submerged them to standard myogenic differentiation medium, and stained for the myogenic marker myosin heavy chain (MF20) (fig. S3C). While control cells differentiated normally and fused to form myotubes, $TRF2^{MuSC\text{-}cKO}$ cells had lower differentiation abilities and did not fuse as readily (figs S3D-E), suggesting impediment of myogenic commitment, which is in accordance with the lack of differentiated fibers we observed post-injury *in vivo* (Fig. 3D). Altogether, these findings indicate that TRF2 deletion in MuSCs incapacitates myogenic commitment.

Deletion of TRF2 affects stem cell identity related to myogenesis and no other lineages.

We investigated whether lack of TRF2 compels cells to reprogram into other non-myogenic lineages commonly found among diseased muscles, which could account for the reduced detection of MuSC-specific signatures and/or reduced detectable cell numbers (Fig. 4A and fig. S2J-M). However, we found no evidence of an increased expression in either fibrogenic, adipogenic, neurogenic, or osteogenic markers in $TRF2^{MuSC\text{-}cKO}$ MuSCs (fig. S5C), indicating that cell type conversion to these lineages is unlikely. In contrast, myogenic gene expressions of MuSCs at both uninjured and 3 DPI displayed downregulation of committed MuSCs markers in $TRF2^{MuSC\text{-}cKO}$ MuSCs (fig. S5E-F), further demonstrating that the absence of TRF2 abrogates stem cell identity associated to myogenic characteristics. Furthermore, gene set enrichment analysis (GSEA) illustrates that stem cell differentiation, muscle cell development, and myoblast differentiation genes are dysregulated in TRF2 deficient MuSCs (fig. S5C-D), in agreement with the differentiation defects we observed in injured tissues (Fig. 3D-E).

TRF2 loss impairs MuSC activation without causing senescence or proliferative arrest.

In some stem-cell contexts, loss of TRF2 has been linked to proliferative arrest and senescence(*18*), a state where a cell permanently exits the cell cycle and stops dividing. We therefore next investigated whether $TRF2^{MuSC\text{-}cKO}$ MuSCs show impaired proliferative capacity and/or evidence of senescence. To assess spontaneous activation from quiescence, EdU was administered between 3-14 days after tamoxifen-induced deletion of TRF2 in MuSCs (fig. S6A). We did not observe an increase in EdU^{+}/$mCherry^{+}$ MuSCs in $TRF2^{MuSC\text{-}cKO}$ mice compared to controls (fig. S6A-C), indicating that TRF2 loss does not trigger premature or inappropriate functional activation, despite reduced transcriptomic quiescence markers. We next assessed the kinetics of MuSC activation by measuring the time to initiate DNA replication by measuring first EdU incorporation following an activation stimuli. $TRF2^{MuSC\text{-}cKO}$ MuSCs exhibited a significant delay in EdU incorporation compared to controls (fig. S6D-E), indicating impaired entry into the cell cycle from quiescence. By contrast, once activated, TRF2-deficient MuSCs displayed normal proliferation kinetics, as the time to EdU incorporation in already activated MuSCs was

comparable to controls (fig. S6F-G), in line with our previous data (Fig. 3). Thus, the primary defect in TRF2-deleted MuSCs lies in entry into the myogenic program and fate output, rather than in bulk cycling after activation, consistent with our *in vivo* proliferation assay (Fig. 3G). Together, these findings show that although TRF2 ablation reduces transcriptomic markers of quiescence, it does not induce functional quiescence exit. Instead, TRF2 loss disrupts the proper activation process of MuSCs, leading to delayed cell-cycle entry while preserving proliferative capacity after activation. TRF2 is therefore required to maintain a competent quiescent state that enables timely activation, rather than simply enforcing quiescence per se. We next performed senescence-associated β-galactosidase (SA-β-gal) staining on freshly isolated MuSCs and observed no differences in the proportion of SA-β-gal^{+} cells between control and TRF2$^{MuSC\text{-}cKO}$ MuSCs (fig S7A-B). Moreover, we found no evidence of upregulation in the expression levels of established senescence-associated genes such as *p16*, *p53*, or *p57* in TRF2$^{MuSC\text{-}cKO}$ MuSCs, while *p16* and *p57* were even downregulated (fig. S7C). These assays do not support increased senescence in TRF2-deficient quiescent MuSCs. Altogether, these results suggest that the stem cell defects caused by TRF2 deletion is neither due to proliferation arrest nor cellular senescence. Instead, TRF2 ablation selectively compromises the activation process, without influencing the proliferation capacity of activated MuSCs.

TRF2 via its non-telomeric *cis*-binding influences Pax7 expression in MuSCs

CUT&Tag analysis revealed that TRF2 is specifically enriched at an ENCODE-annotated Pax7 enhancer, located downstream of the transcription termination site associated with the *Pax7* gene (Fig. 7B). To confirm *Pax7* as a target gene of TRF2, we isolated MuSCs and measured the levels of this transcription factor by quantitative RT-PCR. This analysis showed a significant reduction of *Pax7* expression in TRF2$^{MuSC\text{-}cKO}$ MuSCs (fig. S16A), which aligns with our RNAseq data (Fig. 4A) and further endorses that TRF2 via its non-telomeric *cis*-binding, influences *Pax7* gene expression in MuSCs.

Supplementary Figures

Supplementary Fig. 1

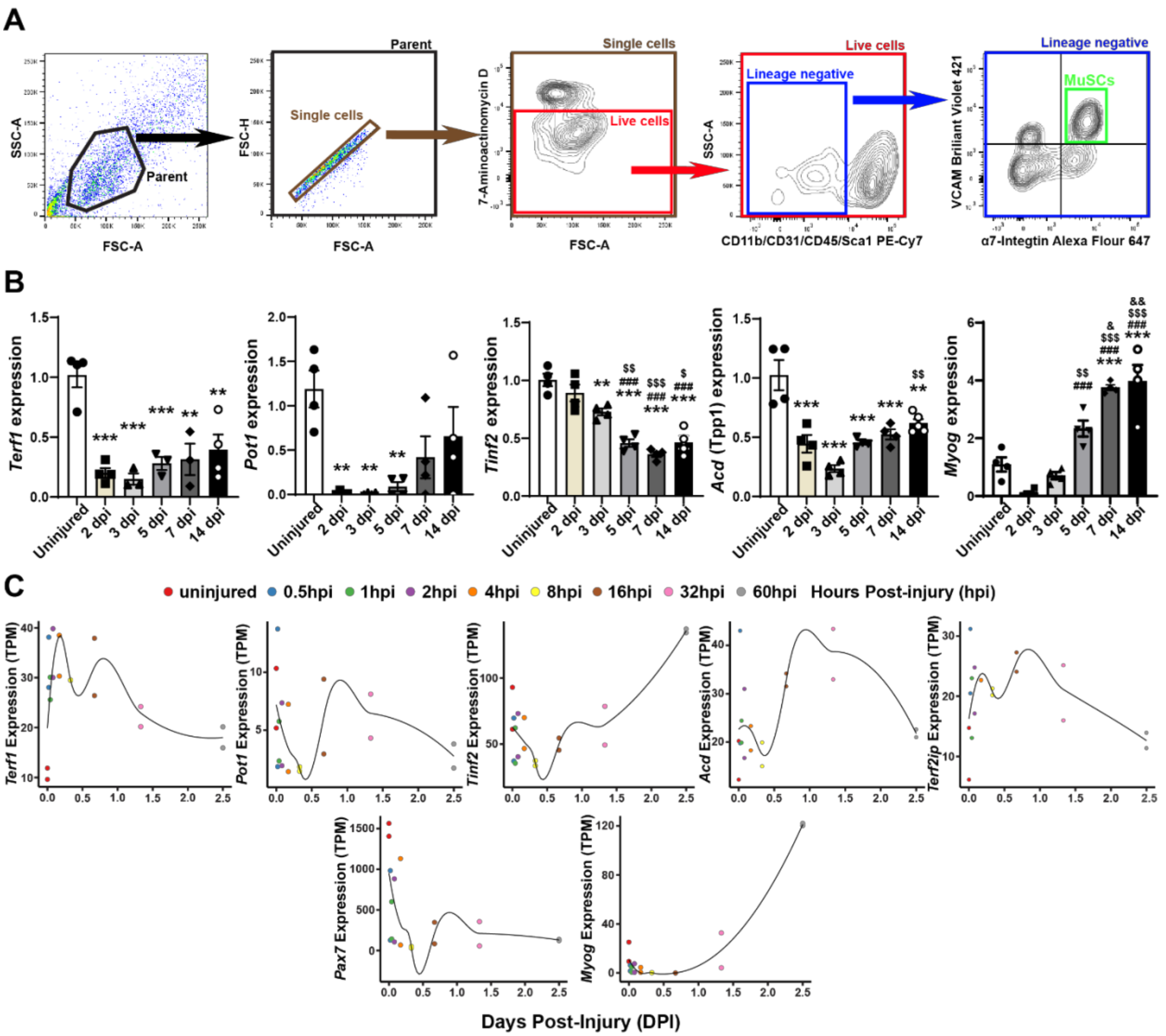

Fig S1. Expression of *Terf2* and other genes in MuSCs.

(A) Schematic of muscle stem cell (MuSC) isolation from skeletal muscles by fluorescent-activated cell sorting (FACS). Gating strategy of parent population (black box) of muscle cell isolation, gating of single cells (brown box), and within single cells, we select only live cells (7-AAD negative, red box). MuSC enrichment within live cells is accomplished by gating CD11b$^-$/CD45$^-$/CD31$^-$/Sca1$^-$ (lineage negative, blue box) populations followed by gating for double positives (VCAM$^+$/α7-integrin$^+$, green box). **(B)** Gene expression analysis of shelterin components, including *Terf1*, *Pot1*, *Tinf2* (TIN2), and *Acd* (TPP1), in MuSCs isolated at the indicated time points after injury. *MyoG* expression is shown as a positive control for regenerative myogenic progression. *Gapdh* and *Tbp* were used as endogenous control. n= 3-4 mice (3 months old) per group. **(C)** Analysis of a published dataset (GSE189074) showing expression profiles of shelterin components [Terf1, Pot1, Tinf2 (TIN2), Acd (TPP1), and Terf2ip (Rap1)] and myogenic

genes (Pax7 and MyoG). n = 2 per group. Data are presented as mean ± SEM. Statistical analysis was performed using one-way ANOVA with Tukey's multiple-comparison test. **P < 0.01 and ***P < 0.001 versus control; ###P < 0.001 versus 2 DPI; $P < 0.05, $$P < 0.01, and $$$P < 0.001 versus 3 DPI; &&P < 0.01 versus 5 DPI.

Supplementary Fig. 2

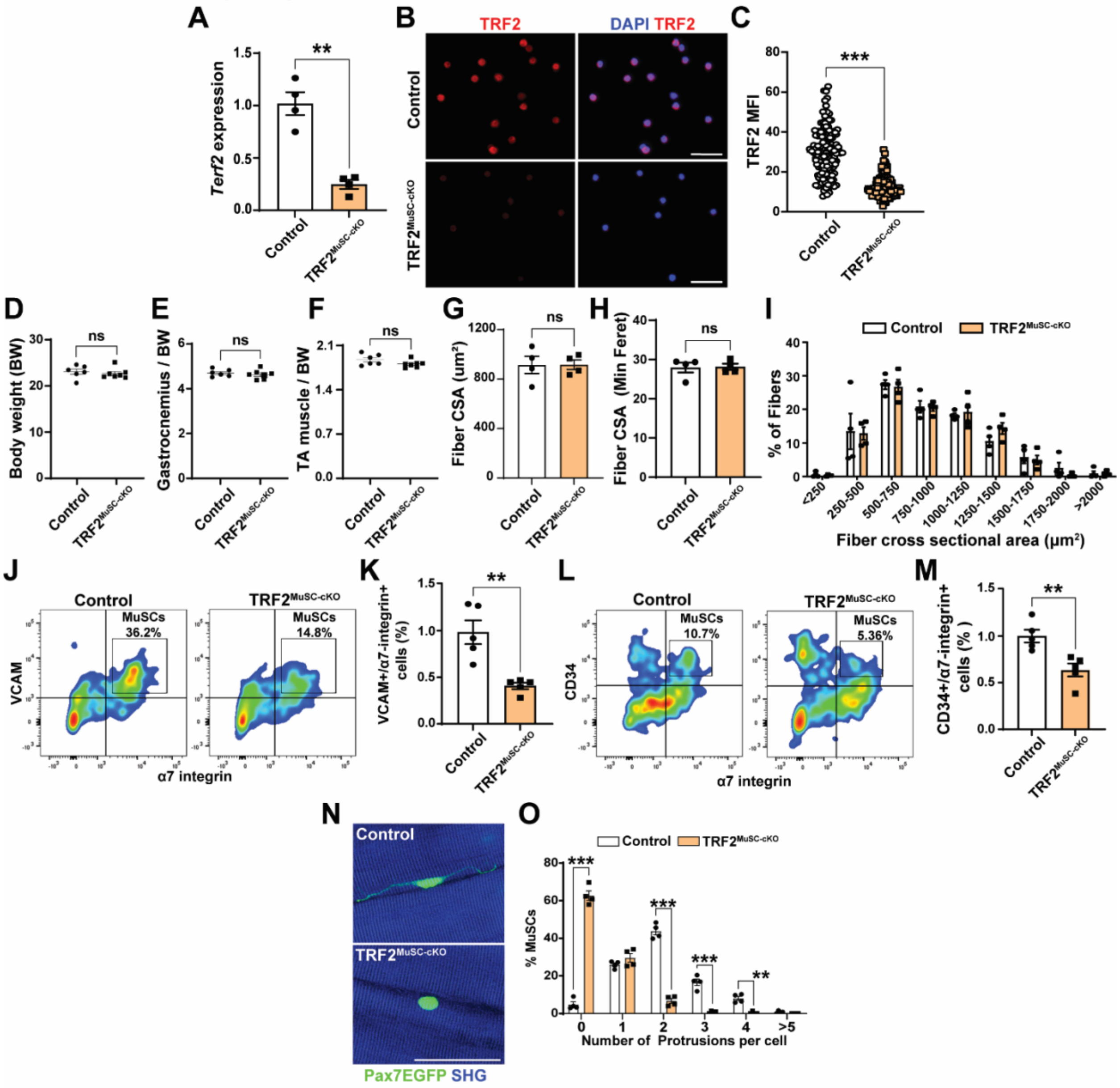

Fig S2. TRF2 ablation disturbs MuSC quiescence and reduces MuSC population of uninjured TRF2$^{MuSC\text{-}cKO}$ muscles.

(A) Quantitative RT-PCR analysis confirming efficient *Terf2* deletion following tamoxifen administration. *Gapdh* and *Tbp* were used as endogenous control (n=4 mice per genotype). **(B)** Representative immunofluorescence images showing reduced TRF2 protein level in TRF2$^{MuSC\text{-}cKO}$ MuSCs compared with controls. Scale bar, 20µm. **(C)** Quantification of TRF2 mean fluorescence intensity (MFI) from (**B**). N=100 cells per genotype from 3 independent biological replicates. **(D)** Body weight (BW) of control and TRF2$^{MuSC\text{-}cKO}$ mice (n=6 mice per genotype). **(E and F)** Gastrocnemius (**E**) and Tibialis anterior (**F**) muscle mass normalized to body weight (n = 6 mice per genotype). **(G)** Fiber cross-sectional area (CSA) in µm^2 of uninjured control and TRF2$^{MuSC\text{-}cKO}$ muscle (n=4 mice per genotype). **(H)** Fiber CSA measured by minimum Feret diameter (n=4

mice per genotype). **(I)** Distribution of myofiber CSA between genotypes (n=4 mice per genotype). **(J)** Representative flow cytometry plots showing MuSCs identified as VCAM+/α7-integrin+ cells. **(K)** Quantification of MuSC frequency from (J) (n=5 mice per genotype). **(L)** Representative flow cytometry plots showing MuSCs identified as CD34+/α7-integrin+ cells. **(M)** Quantitation of MuSC frequency from (L) (n=5 mice per genotype). **(N)** Representative two-photon microscopy images showing MuSCs morphology in uninjured control and TRF2$^{MuSC\text{-}cKO}$ muscles. Scale bar, 50µm. **(O)** Quantification of MuSCs with different numbers of cytoskeletal protrusions (n=4 mice per genotype). Data are presented as means ±SEM. Statistical analysis was performed using two-tailed unpaired t tests with Welch's correction in (A) and (C–F), and two-way ANOVA with Bonferroni's multiple-comparison test in (I) and (O). $^{**}P < 0.01$; $^{***}P < 0.001$; n.s., not significant.

Supplementary Fig. 3

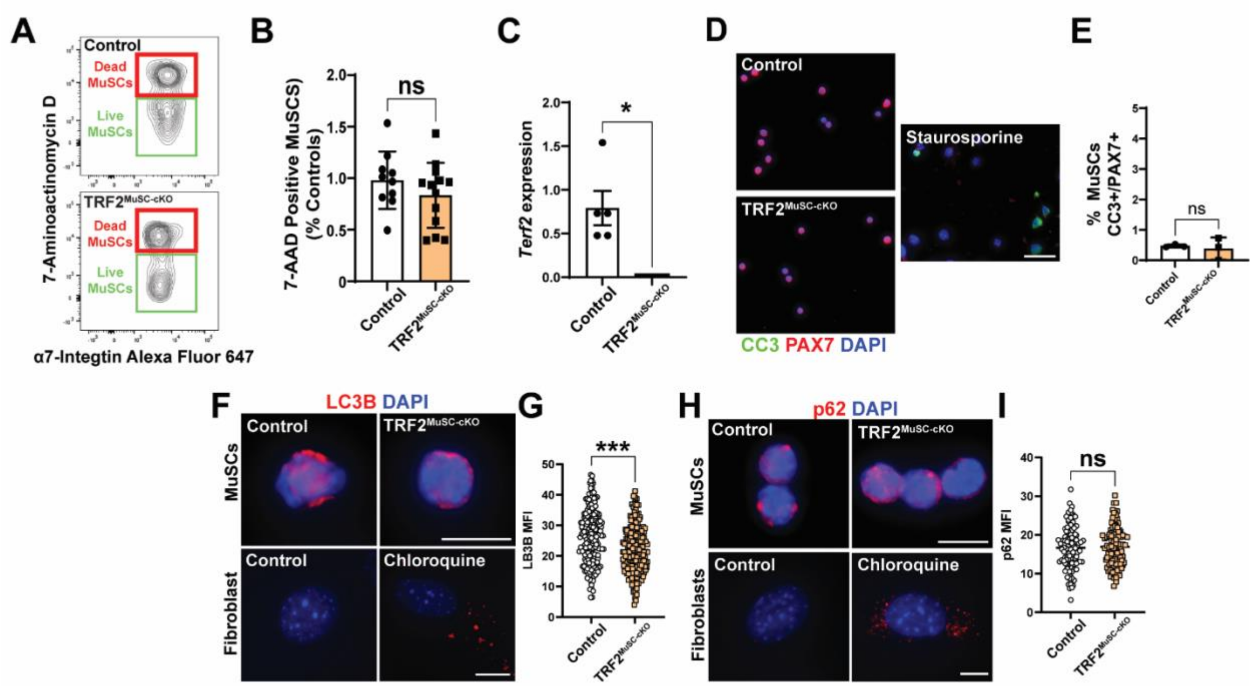

Fig S3. Reduction of the MuSCs population in TRF2$^{MuSC\text{-}cKO}$ muscle is independent of cell death.

(**A**) Representative flow cytometry contour plots from controls (top) and TRF2$^{MuSC\text{-}cKO}$ MuSCs (bottom) showing dead cells (red rectangle), and live cells (green rectangle) based on 7-Aminoactinomycin D (7-AAD) staining. **(B)** Quantification of 7-AAD^{+} MuSCs (n=10-13 mice per genotype). (**C**) Quantitative RT-PCR analysis confirming efficient *Terf2* deletion in MuSCs following 4-hydroxytamoxifen (4-OHT) treatment. n = 5 independent biological replicates per genotype. (**D**) Representative immunofluorescence images of control and TRF2$^{MuSC\text{-}cKO}$ MuSCs stained for cleaved caspase 3 (CC3), DAPI (nuclei) and Pax7. Staurosporine-treated cells served as a positive control. Scale bar, 20µm. (**E**) Quantification of CC3+/Pax7+ MuSCs (n=3 mice per genotype). (**F**) Representative immunofluorescence images of the autophagic marker LC3B in control (top) and TRF2$^{MuSC\text{-}cKO}$ MuSCs (bottom). Fibroblasts treated with 50 µM chloroquine served as a positive control. Scale bar, 4µm. (**G**) Quantification of LC3B mean fluorescence intensity (MFI) from (**F**). (**H**) Representative immunofluorescence images of the autophagic flux marker p62 in control and TRF2$^{MuSC\text{-}cKO}$ MuSCs. Fibroblasts treated with 50 µM chloroquine served as a positive control. Scale bar, 4µm. (**I**) Quantification of p62 mean fluorescence intensity (MFI) from (**H**). N=100 cells from 4 independent biological replicates. Data are presented as means ±SEM. Statistical analysis was performed using two-tailed unpaired t-test with Welch's correction in (B-C), (E), (G), and (I). *P<0.05; *** P<0.001; ns represents not significant.

Supplementrary Fig. 4

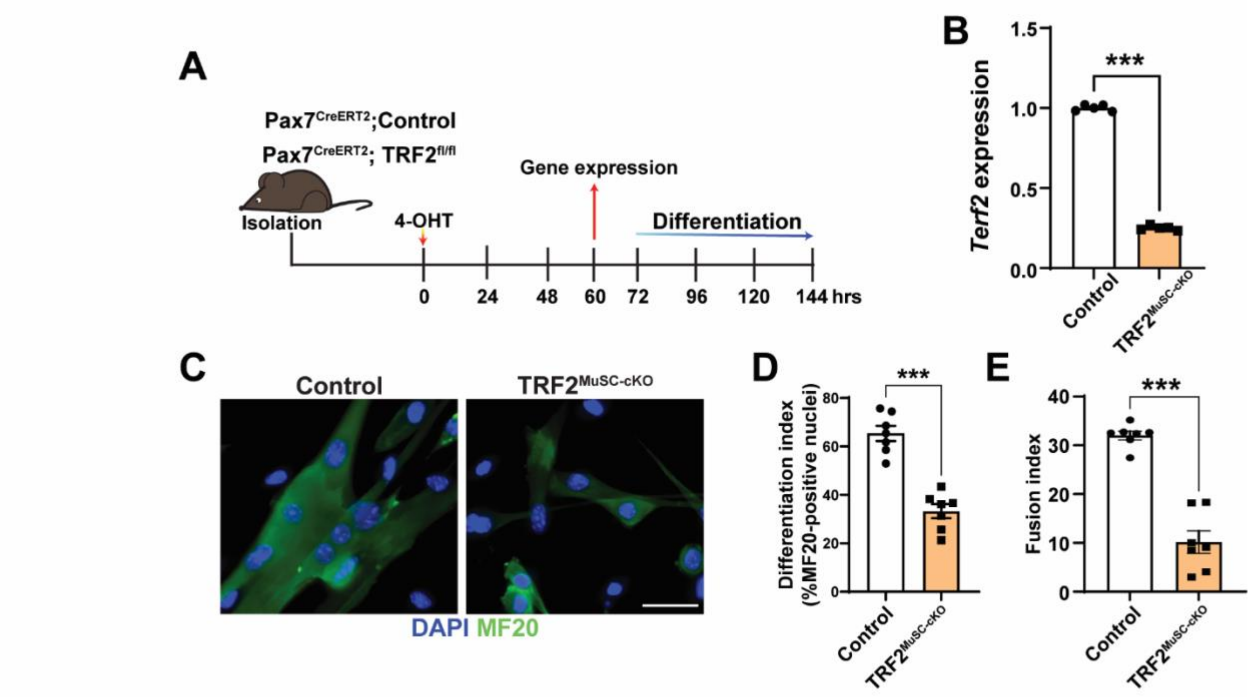

Fig S4. TRF2 is required for efficient myogenic commitment of activated MuSCs.

(A) Experimental design for 4-hydroxytamoxifen (4-OHT) treatment of isolated MuSCs, followed by submersion in differentiation media. **(B)** Quantitative RT-PCR analysis confirming efficient *Terf2* deletion following 4-OHT treatment (n = 5 biological replicates per genotype). **(C)** Representative images of differentiated control and TRF2$^{MuSC\text{-}cKO}$ cells stained for the differentiation marker myosin heavy chain (MF20) and nuclei (DAPI). Scale bar, 20μm. **(D)** Differentiation index, defined as the percentage of MF20+ cells relative to total nuclei (n=7 biological replicates per genotype). **(E)** Fusion index, defined as percentage of MF20+ myotubes containing 3 or more nuclei relative to the total nuclei (n=7 biological replicates per genotype). Data are represented as means ±SEM. Statistical analysis was performed using two-tailed unpaired t-test with Welch's correction in (B), (D, and (E). *** P<0.001.

Supplementary Fig. 5

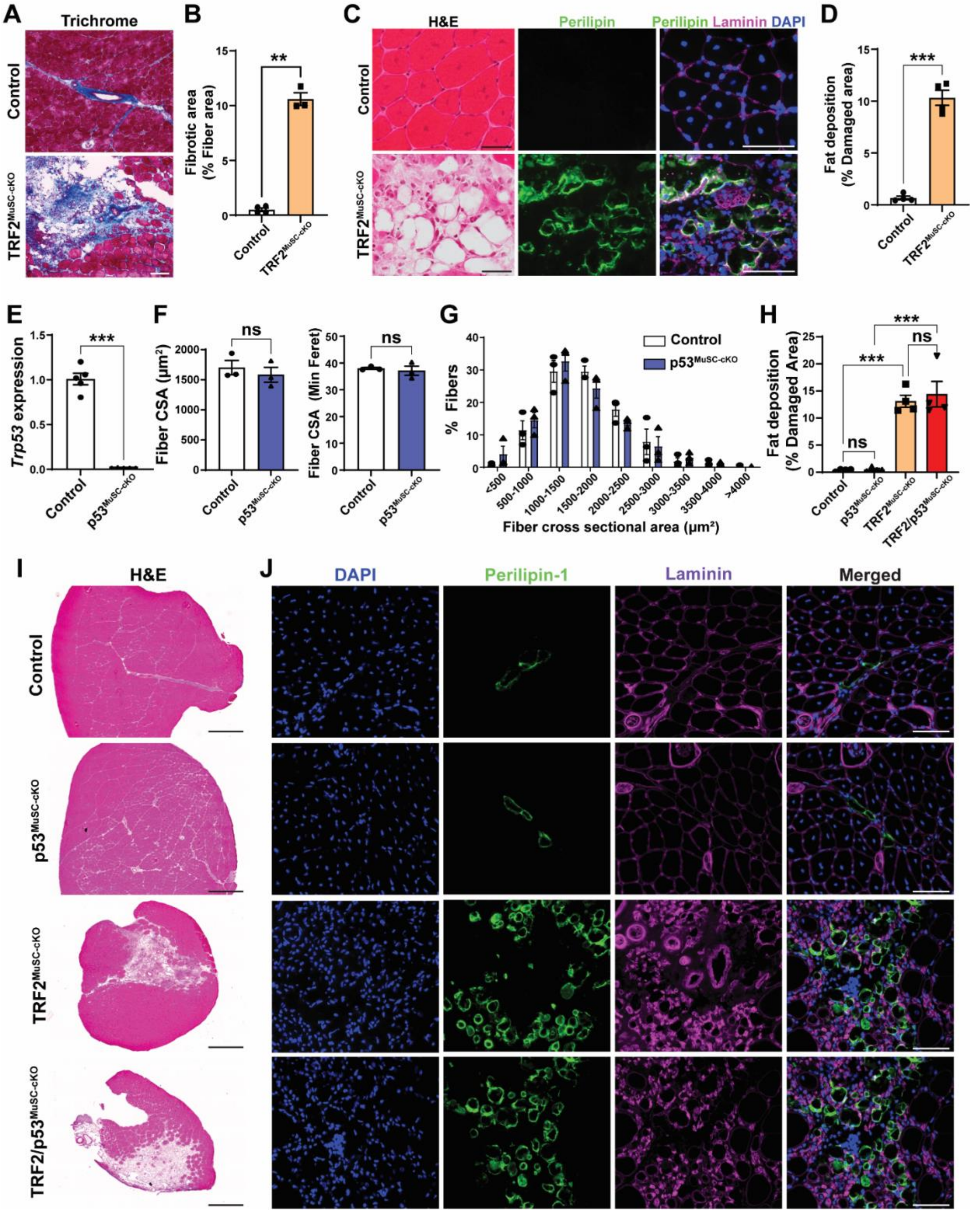

Fig S5. Increased tissue damage and adipose tissue accumulation in regenerating TRF2$^{MuSC-cKO}$ muscles.

(A) Representative Masson's trichome staining of tibialis anterior (TA) muscles from control and TRF2$^{MuSC-cKO}$ mice at 14 days post-injury (DPI). Scale bar, 100µm. **(B)** Quantification of fibrotic area in control and TRF2$^{MuSC-cKO}$ muscles (n=3 per genotypes). **(C)** Representative H&E staining (left; scale bar, 20µm) and immunofluorescence staining for perilipin (lipid droplets), DAPI (nuclei), and laminin (fibers) (middle and right; scale bar, 50µm) in control and TRF2$^{MuSC-cKO}$ muscles at 14 DPI. **(D)** Quantification of adipose tissue area in control and TRF2$^{MuSC-cKO}$ muscles at 14 DPI (n=4 per genotype). **(E)** Quantitative RT-PCR analysis confirming efficient MuSC-specific p53 deletion following tamoxifen administration (n = 4 mice per genotype). **(F)** Myofiber cross-sectional area (CSA) in µm^2 in uninjured control and p53^{MuSC-} muscles (left) and myofiber area measured by minimum Feret diameter (right) (n=3 mice per genotype). **(G)** Fiber size distribution in control and p53$^{MuSC-cKO}$ mice at 14 DPI. **(H)** Quantification analysis of adipose tissue area in control, p53$^{MuSC-cKO}$, TRF2$^{MuSC-cKO}$, and TRF2/p53$^{MuSC-cKO}$ muscles at 14 DPI (n=4 per genotype). **(I)** Representative H&E staining of regenerating muscles from the indicated genotypes at 14 DPI. Scale bar, 500µm. **(J)** Representative immunofluorescence images stained for DAPI (nuclei), Perilipin-1 (lipid droplets), Laminin (fiber) at 14DPI. Scale bar, 50µm. Data are presented as means ±SEM. Statistical analysis was performed using two-tailed unpaired t-test with Welch's correction in (B), (D-F), and two-way ANOVA test with Tukey's correction in (G) and (H). **P<0.01; ***P<0.001; ns, not significant.

Supplementary Fig. 6

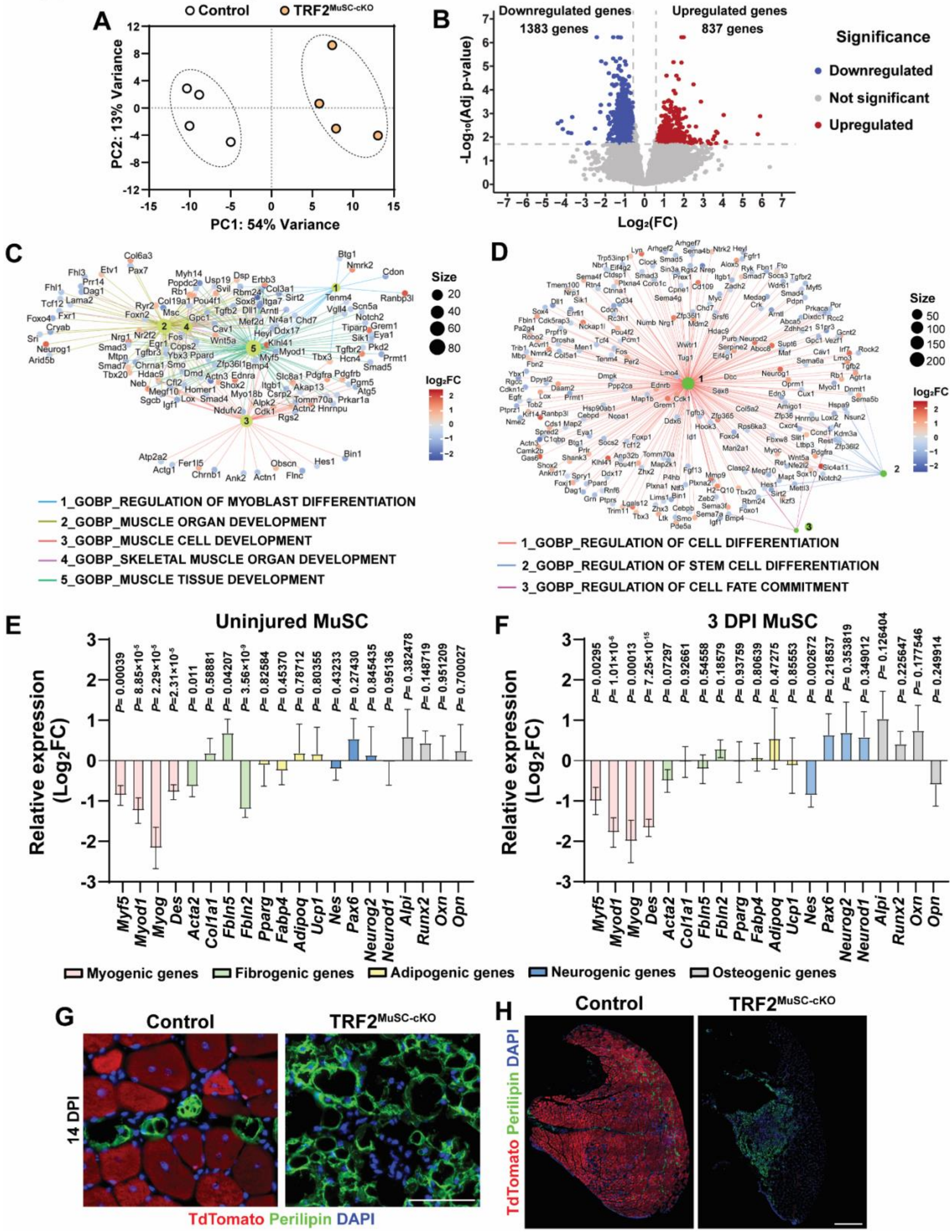

Fig S6. TRF2 preserves the myogenic transcriptional program of MuSCs without promoting alternative lineage conversion.

A) Principal component analysis (PCA) of transcriptomes from freshly isolated MuSCs obtained from uninjured control and TRF2$^{MuSC\text{-}cKO}$ muscles. **(B)** Volcano plot showing differentially expressed genes in TRF2$^{MuSC\text{-}cKO}$ MuSCs relative to controls. 1383 genes were downregulated, and 837 genes were upregulated. **(C)** Gene Ontology (GO) network analysis highlighting significantly downregulated pathways associated with myogenic differentiation and muscle development in quiescent TRF2$^{MuSC\text{-}cKO}$ MuSCs. **(D)** GO network analysis highlighting significantly downregulated pathways associated with cell differentiation, stem cell differentiation, and cell fate commitment in quiescent TRF2$^{MuSC\text{-}cKO}$ MuSCs. **(E** and **F)** Gene expression analysis of lineage-specific markers. Expression of fibrogenic, adipogenic, neurogenic and osteogenic genes was largely unchanged, whereas myogenic genes (*Myf5*, *Myod1*, *Myog*, *Des*) were significantly reduced in MuSCs isolated from (**E**) uninjured and (**F**) injured muscles at 3 days post-injury (3 DPI). n=3-4 mice (3-month-old) per genotype. Data are presented as means ±SEM. Exact P values are indicated in the graphs. (**G**) Representative immunofluorescence images of regenerating muscles at 14 DPI from control and TRF2$^{MuSC\text{-}cKO}$ mice stained for tdTomato (red), perilipin (green), and DAPI (blue). Scale bar, 50µm. (**H**) Representative immunofluorescence images of regenerating muscles at 14 DPI stained for tdTomato (red), perilipin (green), and DAPI (blue). Note the lack of tdTomato expression in perilipin-positive adipocytes within the TRF2$^{MuSC\text{-}cKO}$ injured areas. Scale bar, 500µm.

Supplementary Fig. 7

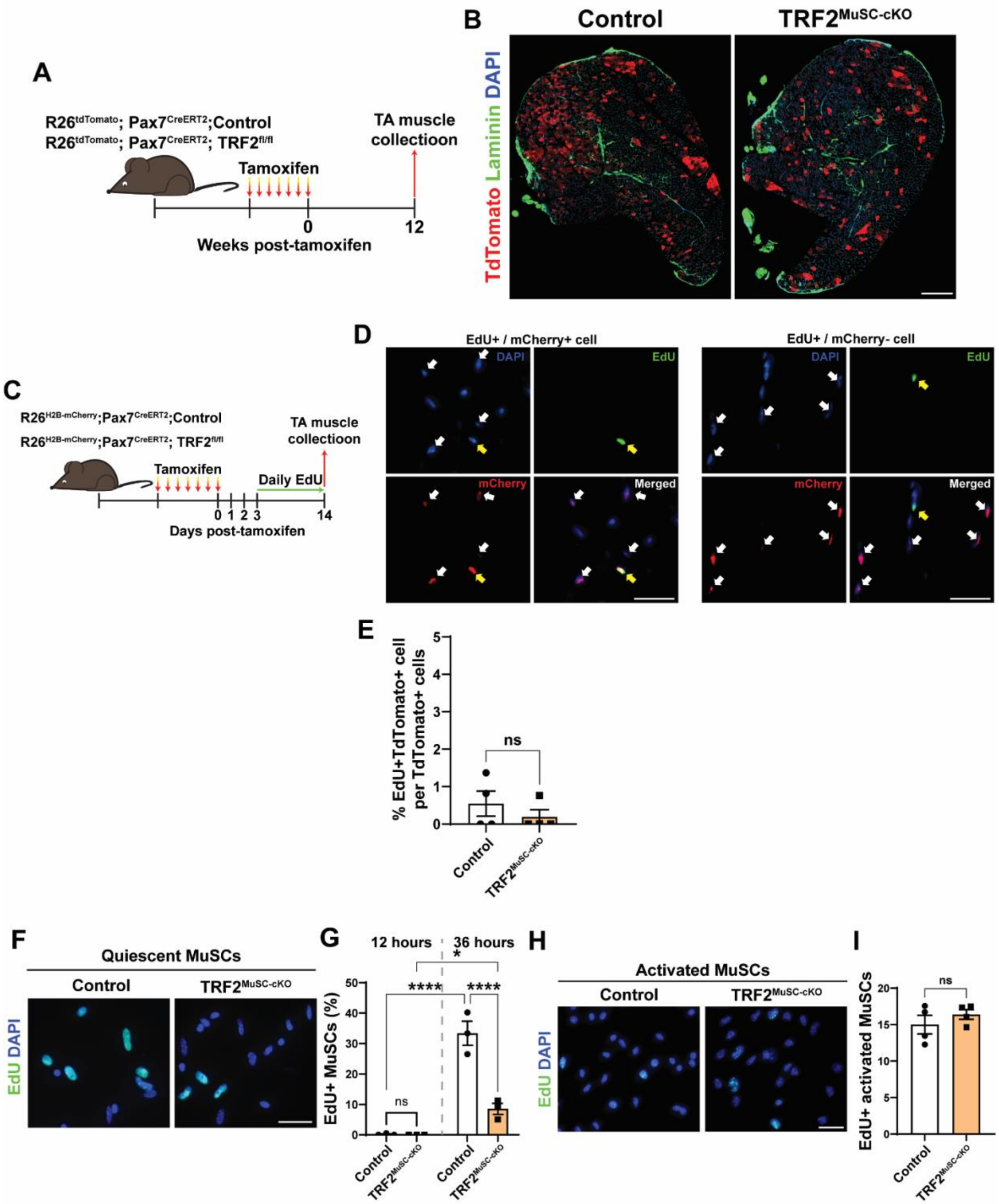

Fig S7. TRF2 deletion alters MuSC fate transition while preserving proliferative capacity in activated MuSCs.

(A) Experimental scheme for lineage tracing in control and TRF2-deficient Pax7-lineage mice carrying the *R26*^{tdTomato} reporter. **(B)** Representative images of tibialis anterior (TA) muscle sections from control and TRF2$^{MuSC\text{-}cKO}$ mice stained for tdTomato, laminin, and DAPI. Scale bar, 500µm. **(C)** Experimental design for lineage-tracing in control and TRF2-deficient Pax7$^{H2B\text{-}mCherry}$ mice followed by daily EdU administration. **(D)** Representative images of EdU, mCherry, and DAPI staining in muscle sections. White arrows indicate mCherry^{+} cells and yellow arrows mark EdU^{+}/mCherry^{+} cells. Scale bar, 20µm. **(E)** Quantification of EdU^{+}/mCherry^{+} cells as a percentage of total mCherry^{+} cells in control and TRF2-deficient muscles. **(F)** Representative images of quiescent MuSCs stained for EdU and DAPI (nuclei). Scale bar, 20µm. **(G)** Quantification of EdU incorporation in quiescent MuSCs after 12 and 36 hours in isolation/culture. **(H)** Representative images of activated MuSCs stained for EdU and DAPI (nuclei). Scale bar, 20µm. **(I)** Quantification of EdU incorporation in activated MuSCs from control and TRF2$^{MuSC\text{-}}$cKO mice. More than 100 cells from n=3 mice per genotype were analyzed. Data are presented as means ±SEM. Statistical analysis was performed using two-tailed unpaired t-tests with Welch's correction in (E) and (I), and two-way ANOVA test Bonferroni's multiple-comparison test in (G). *P<0.05; ****P<0.0001; ns, not significant.

Supplementary Fig. 8

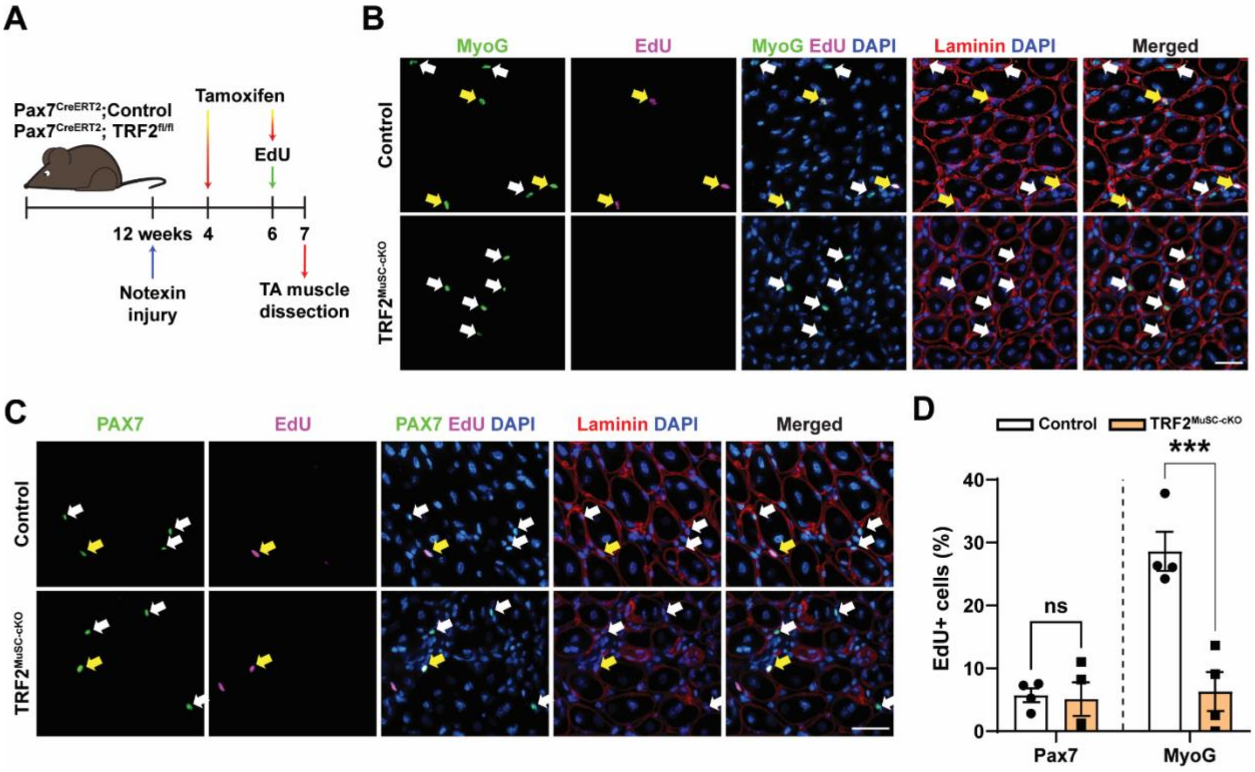

Fig S8. TRF2 ablation uncouples proliferation from myogenic commitment.

(A) Experimental design for acute muscle injury and EdU labeling in Control and TRF2$^{MuSC\text{-}cKO}$ mice. **(B)** Representative images of regenerating tibialis anterior (TA) muscle sections stained for MyoG, EdU, laminin, and DAPI. White arrows indicate MyoG$^+$/EdU$^-$, whereas yellow arrows indicate MyoG$^+$/ EdU$^+$ cells. Merged images are shown on the right. Scale bar, 20µm. **(C)** Representative images of regenerating TA muscle sections stained for Pax7, EdU, laminin, and DAPI. White arrows indicate Pax7$^+$/EdU$^-$ and yellow arrows indicate Pax7$^+$/ EdU$^+$ cells. Scale bar, 20µm. **(D)** Quantification of EdU incorporation within Pax7$^+$ and MyoG$^+$ cell populations. The percentage of EdU$^+$/Pax7$^+$ cells was unchanged, whereas the percentage of EdU$^+$/MyoG$^+$ cells was reduced in TRF2$^{MuSC\text{-}cKO}$ muscles compared with controls. Data are presented as means ± SEM. Statistical analysis was performed using two-tailed unpaired t-tests with Welch's correction. ****P < 0.0001; ns, not significant.

Supplementary Fig. 9

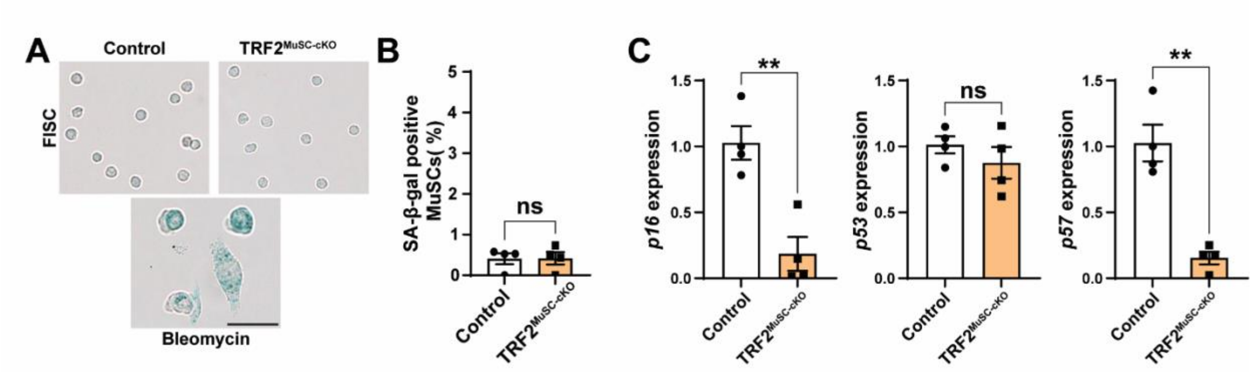

Fig S9. TRF2 loss does not exhibit hallmarks of cellular senescence in MuSC-derived cells.

(**A**) Representative images of freshly isolated MuSCs (top) stained for the senescence marker β-galactosidase (SA-β-gal). Bleomycin-treated myoblasts served as a positive control (bottom). Scale bar, 20μm. (**B**) Quantification of SA-βgal-positive MuSCs in control and $TRF2^{MuSC\text{-}cKO}$ groups. More than 100 cells from 4 independent biological replicates were analyzed. (**C**) Quantitative RT-PCR analysis of the senescence-associated genes *p16*, *p53*, and *p57*, in isolated MuSCs (n=4 mice per genotype). Data are presented as means ±SEM. Statistical analysis was performed using two-tailed unpaired t-tests with Welch's correction in (B) and (C). **P<0.01; ns, not significant.

Supplementary Fig. 10

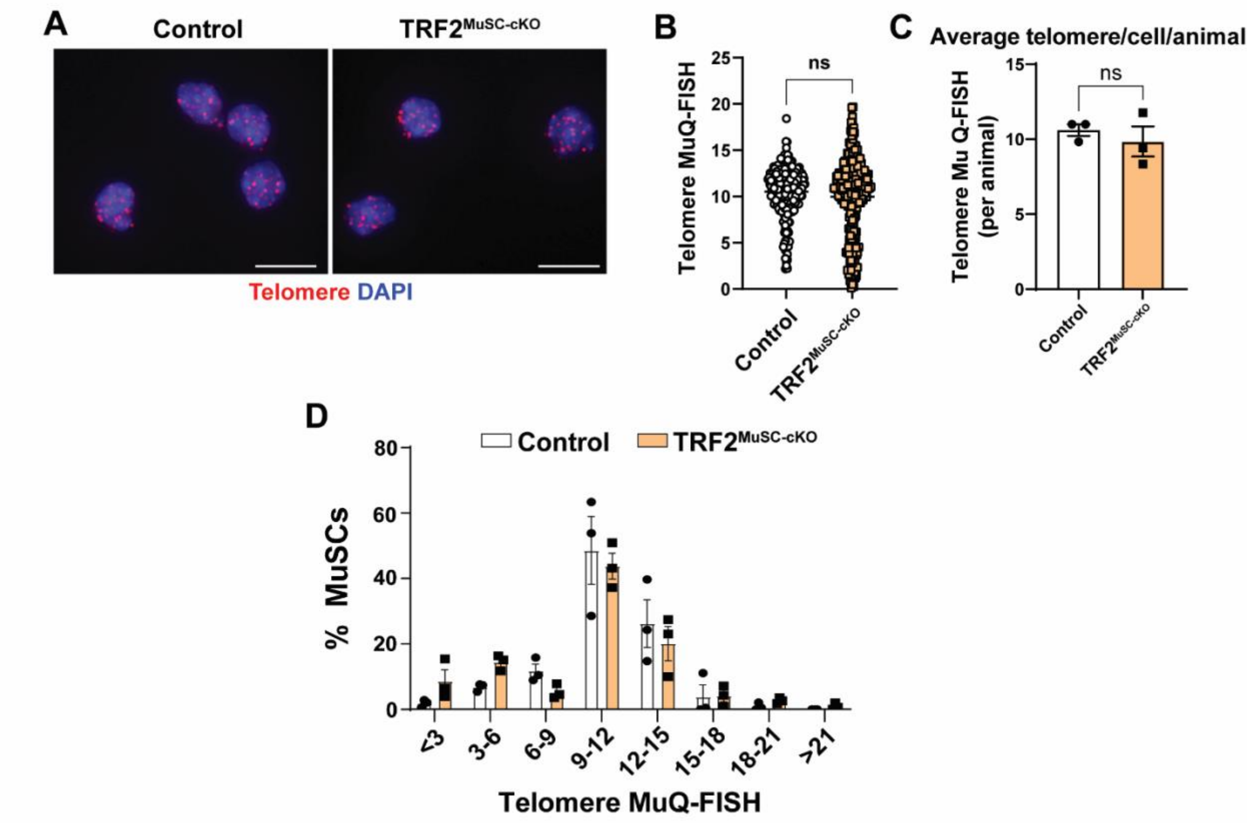

Fig. S10. TRF2 deletion does not alter telomere length in MuSCs.

(**A**) Representative fluorescence in situ hybridization in MuSCs (MuQ-FISH) images showing telomeres and DAPI-stained nuclei in freshly isolated control and TRF2$^{MuSC\text{-}cKO}$ MuSCs. Scale bar, 5µm. (**B**) Quantification of individual telomere fluorescence intensity measured by MuQ-FISH. More than 100 cells from 3 independent biological replicates (3-month-old mice) were analyzed. (**C**) Average telomere fluorescence intensity per cell per animal in Controls and TRF2$^{MuSC\text{-}cKO}$ MuSCs. More than 100 MuSCs per mouse were analyzed from n=3 mice per genotype. (**D**) Distribution of telomere fluorescence intensity (% MuSCs with different telomere length) in control and TRF2$^{MuSC\text{-}cKO}$ MuSCs. More than 100 cells from 3 independent biological replicates (3-months -old mice) were analyzed. Statistical analysis was performed using two-tailed unpaired t-tests with Welch's correction in (B) and (C), and two-way ANOVA with Bonferroni's multiple-comparison test in (D). ns, not significant.

Supplementary Fig. 11

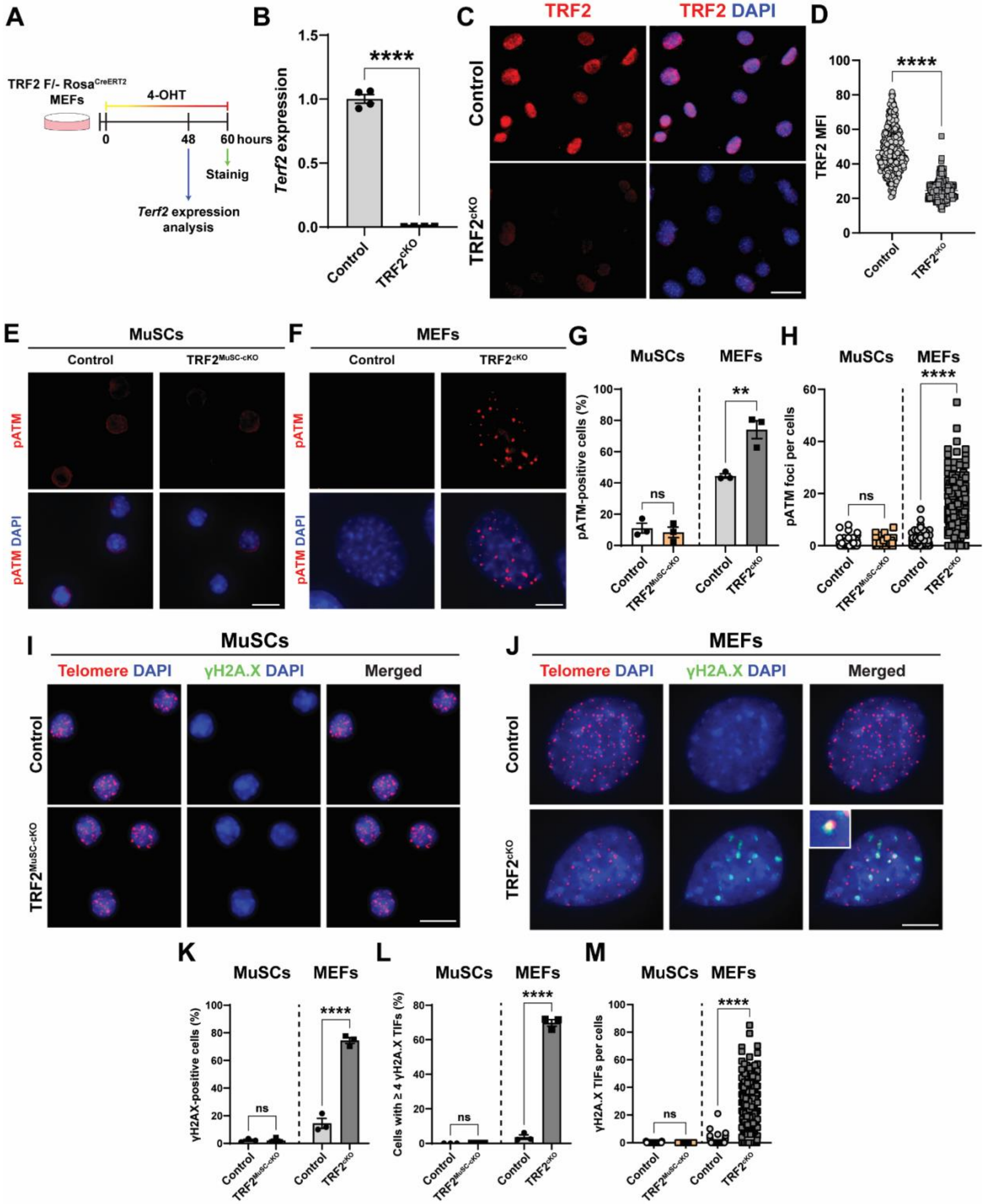

Fig. S11. TRF2 absence triggers DDR activation responses in MEFs but not in MuSCs.

(**A**) Experimental design for *in vitro* TRF2 deletion in mouse embryonic fibroblasts (MEFs). (**B**) Quantitative RT-PCR analysis of *Terf2* expression in control and TRF2^{cKO} MEFs. More than 100

cells from 3 independent replicates were analyzed. **(C)** Representative immunofluorescence images of control and TRF2^{cKO} MEFs stained for TRF2 and DAPI. Scale bar, 20μm. **(D)** Quantification of TRF2 mean fluorescence intensity (MFI) in control and TRF2^{cKO} MEFs. More than 100 cells from 3 independent replicates were analyzed. **(E** and **F)** Representative immunofluorescence images of phosphorylated ATM (pATM) in control and TRF2-deficient MuSCs (**E**) and MEFs (**F**). Nuclei were counterstained with DAPI. Scale bar, 5μm. (**G)** Quantification of the percentage of pATM-positive cells in MuSCs and MEFs. (**H**) Quantification of the number of pATM foci per cell in MuSCs and MEFs. n=3 mice per genotypes were examined and more than 100 cells per cell type were analyzed. **(I** and **J)** Representative telomere dysfunction-induced foci (TIF) analysis by telomere FISH combined with γH2A.X immunostaining in MuSCs (**I**) and MEFs (**J**). Insets highlight representative telomere-associated DNA damage foci. Scale bar, 5μm. (**K** to **M**) Quantification of γH2A.X-associated telomere dysfunction, including the percentage of γH2A.X-positive cells (**K**), the percentage of cells containing ≥4 γH2A.X TIFs (**L**), and the number of γH2A.X TIFs per cell (**M**). For MuSC analyses, n=3 mice per genotypes were examined and more than 100 cells per cell type were analyzed. Data are represented as means ±SEM. Statistical analysis was performed using two-tailed unpaired t-tests with Welch's correction in (B) and (D), and two-way ANOVA test with Tukey's multiple-comparison in (G-H) and (K-M). **** P<0.0001; ns, not significant.

Supplementary Fig. 12

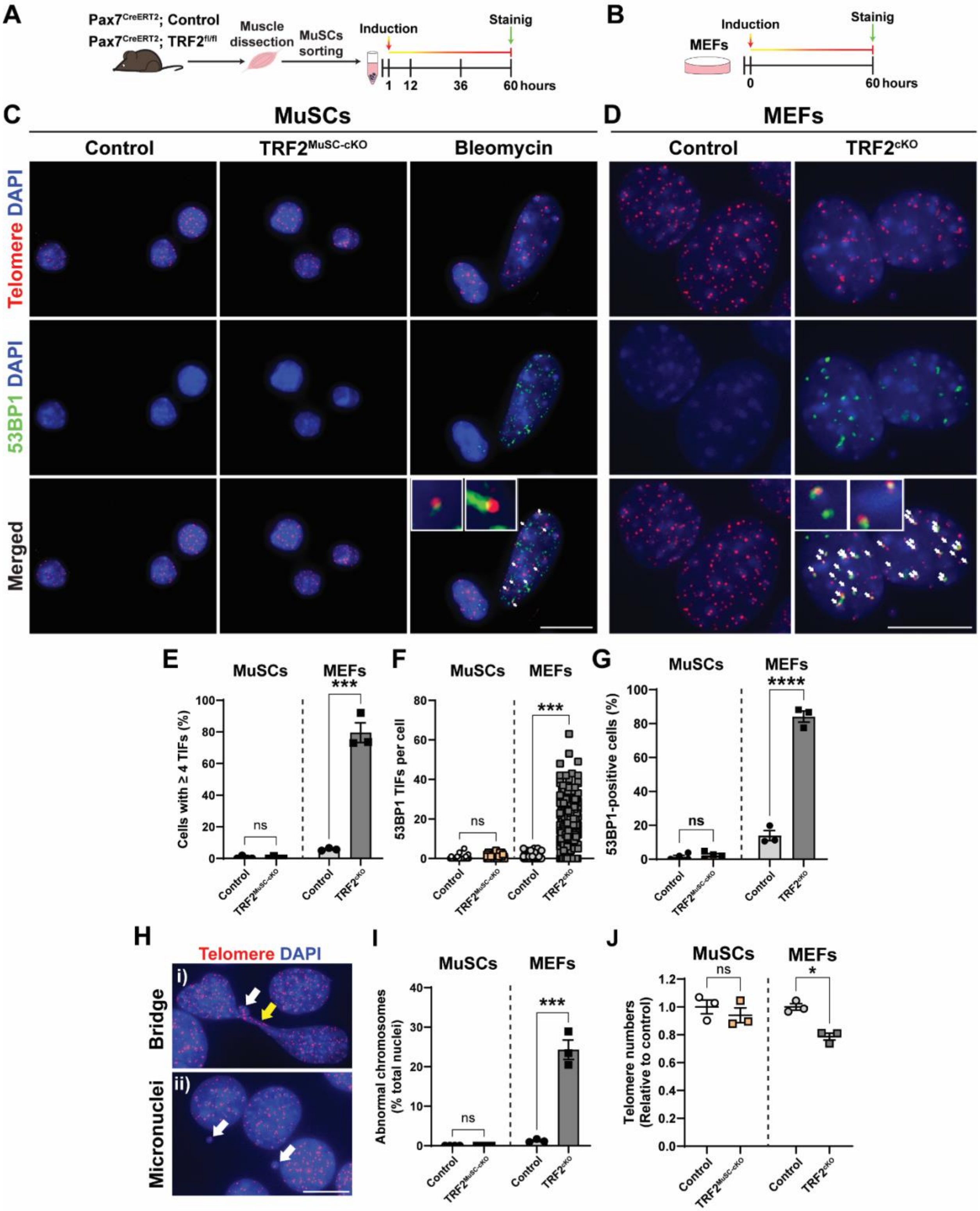

Fig. S12. MuSCs, unlike MEFs, are resistant to TRF2 deletion-induced telomere dysfunction.

(A) Experimental design for analysis of freshly isolated MuSCs. **(B)** Experimental design for analysis of mouse embryonic fibroblasts (MEFs). **(C)** Representative immunofluorescence images

of MuSCs stained for telomeres, 53BP1, and DAPI under control, TRF2-deficient, or bleomycin-treated conditions. Insets highlight representative telomere dysfunction-induced foci (TIFs), identified by colocalization of telomere and 53BP1 signals. Scale bar, 10μm. **(D)** Representative immunofluorescence images of control and TRF2^{cKO} MEFs stained for telomeres, 53BP1, and DAPI. Merged images and representative TIFs are shown in the insets. **(E)** Quantification of cells containing ≥ 4 TIFs in MuSCs and MEFs. **(F)** Quantification of 53BP1-associated TIFs per cell in MuSCs and MEFs. **(G)** Quantification of 53BP1-positive cells in MuSCs and MEFs. More than 100 cells from 3 biological replicates were analyzed for (**E to G**). **(H)** Representative images of abnormal nuclear morphologies following TRF2 deletion, including chromatin bridges (i) and micronuclei (ii). Arrows indicate the abnormalities. **(I)** Quantification of abnormal nuclear morphologies in MuSCs and MEFs. **J)** Quantification of telomere numbers relative to controls in MuSCs and MEFs. Data are presented as means ± SEM. Statistical analysis was performed using two-way ANOVA with Tukey's correction in (E-G) and (I-J). *P < 0.05; **P < 0.001; ***P<0.001; ns, not significant.

Supplementary Fig. 13

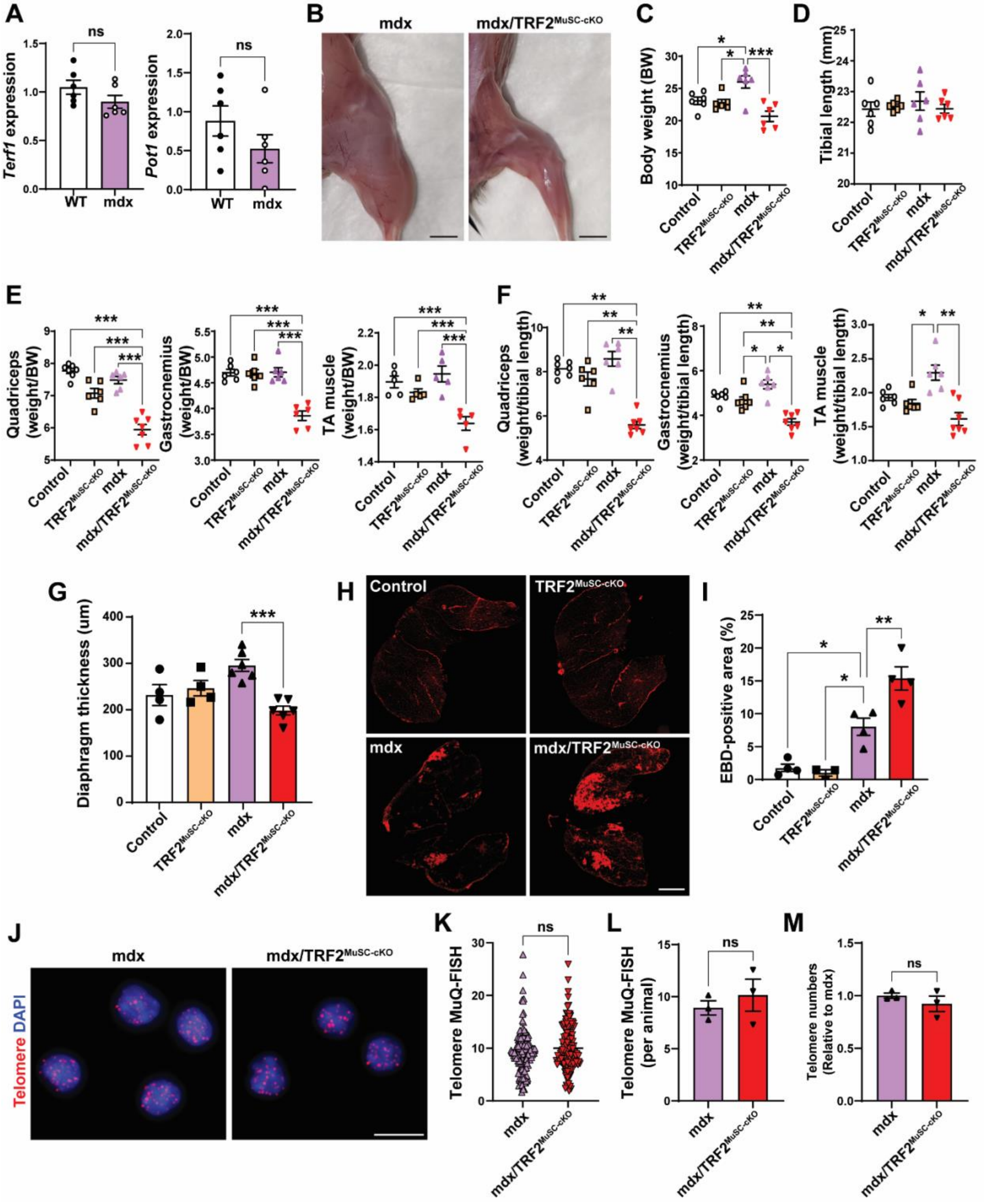

Fig S13. MuSC-specific TRF2 deletion aggravates skeletal muscle atrophy and muscle damage in mdx mice.

(A) Quantitative RT-PCR analysis of the shelterin components *Terf1* and *Pot1* in MuSCs isolated from mdx and TRF2$^{MuSC\text{-}cKO}$ muscles (n=6 per genotype). **(B)** Representative images at 6-month-old animals show atrophied skeletal muscles in mdx/TRF2$^{MuSC\text{-}cKO}$ compared to mdx mice. Scale bar, 5mm. **(C)** Body weight (BW) of controls, TRF2$^{MuSC\text{-}cKO}$, mdx, and mdx/TRF2$^{MuSC\text{-}cKO}$ mice (n=6 mice per genotype; 3 months old). **(D)** Tibial length of the indicated genotypes (n=6 mice per genotype). **(E)** Quadriceps, gastrocnemius, and tibialis anterior (TA) weights normalized to BW (n=6 mice per genotype). **(F)** Quadriceps, gastrocnemius and TA weights normalized o tibial length (n=6 mice per genotype). **(G)** Diaphragm thickness in the indicated genotypes (n=4-6 mice per genotype). **(H)** Representative Evans blue dye (EBD) staining of gastrocnemius muscles. Scale bar, 1mm. **(I)** Quantification of EBD-positive area (%) showing increased damage in mdx/TRF2$^{MuSC\text{-}cKO}$ gastrocnemius muscles (n=4 mice per genotype). (**J**) Representative MuQ-FISH images of telomere and DAPI (nuclei) from freshly isolated mdx and mdx/TRF2$^{MuSC\text{-}cKO}$ MuSCs. Scale bar, 5µm. (**K**) Quantification of individual telomere signal intensity. More than 100 cells from 3 independent biological replicates were analyzed. (**L**) Average telomeres MuQ-FISH per animal in mdx and mdx/TRF2$^{MuSC\text{-}cKO}$ MuSCs. **(M)** Average number of telomere foci per cell in mdx and mdx/TRF2$^{MuSC\text{-}cKO}$ MuSCs. More than 100 MuSCs per mouse were analyzed and n=3 mice per genotype. Data are presented as means ±SEM. Statistical analysis was performed using two-tailed unpaired t tests with Welch's correction in (A) and (K-M), and two-way ANOVA with Tukey's multiple-comparison test in (C-G) and (I). *P<0.05; **P<0.01; ***P<0.001; ns, not significance.

Supplementary Fig. 14

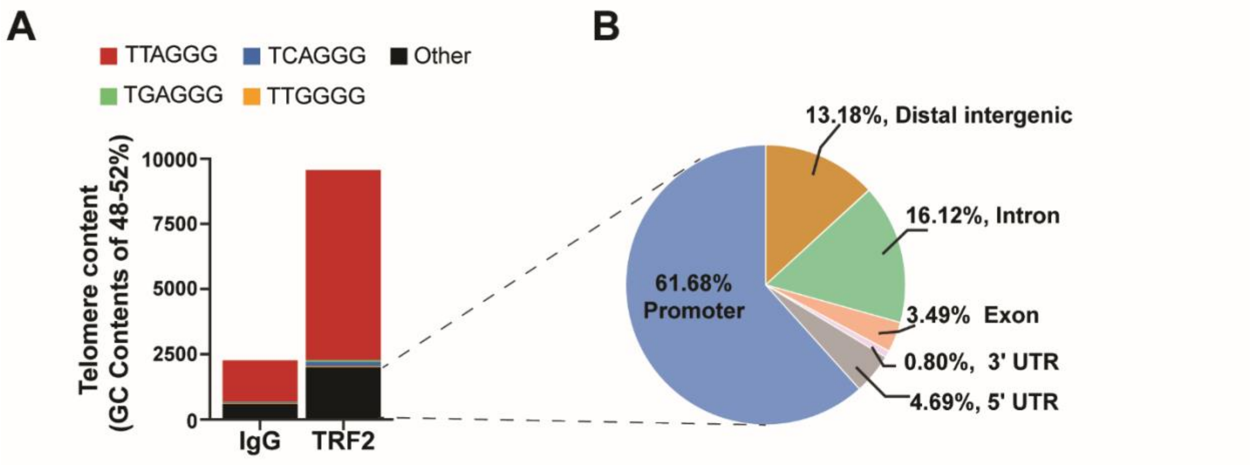

Fig S14. Genome-wide distribution and non-telomeric TRF2-binding sites in MuSCs.

(A) Composition of TRF2-bound DNA sequences. Canonical telomeric repeats (TTAGGG) are shown in red. Telomere variant repeats (TCAGGG, TGAGGG and TTGGGG) are shown in blue, green and yellow, respectively. All remaining sequences are classified as non-telomeric and shown in black. **(B)** Genomic distribution and annotation of non-telomeric TRF2-binding regions identified in MuSCs.

Supplementary Fig. 15

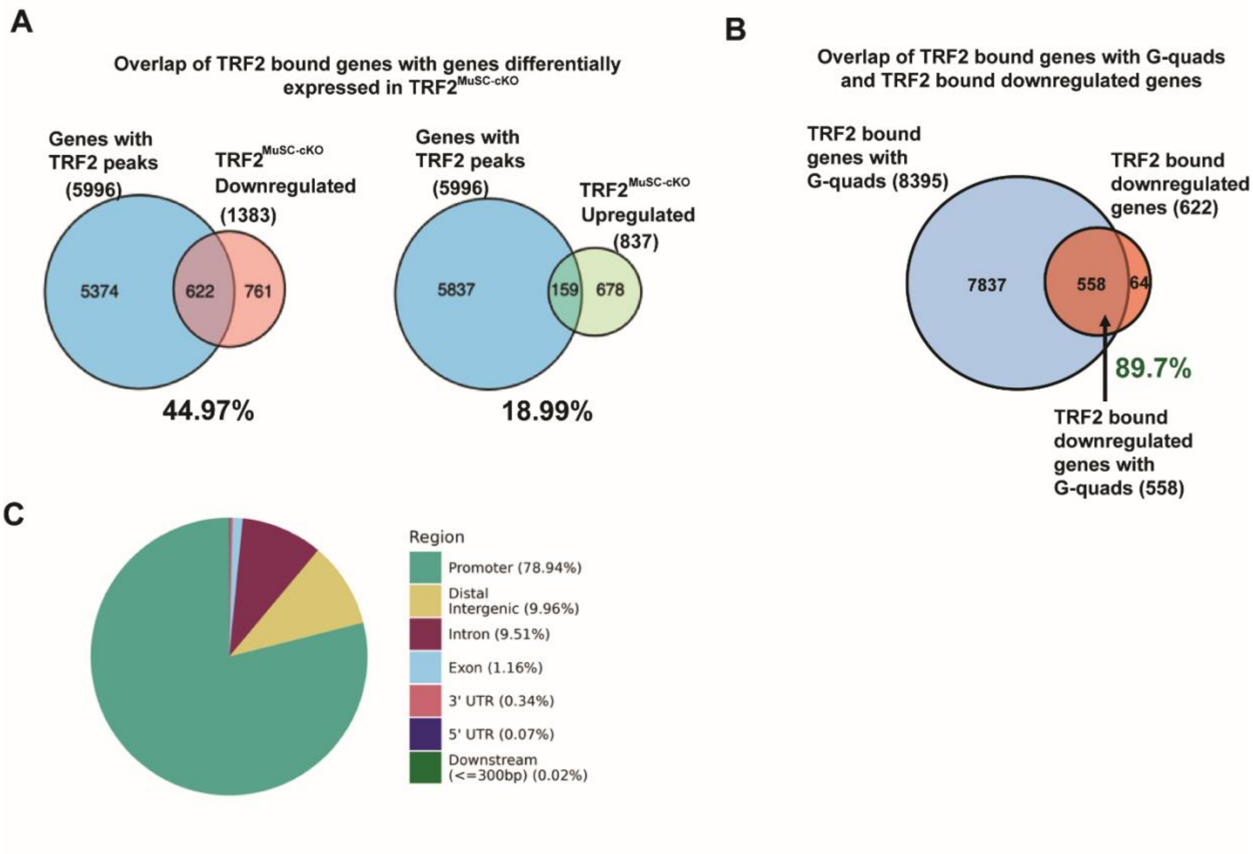

Fig. S15. Integration of TRF2 occupancy, G-quadruplex motifs, and transcriptional regulation in MuSCs.

(A) Overlap between TRF2-bound genes and differentially expressed genes in TRF2$^{MuSC\text{-}cKO}$ MuSCs. (**B**) Overlap among TRF2-bound genes associated with G-quadruplex (G4s) motifs, and TRF2-bound genes that are downregulated in TRF2$^{MuSC\text{-}cKO}$ MuSCs. (**C**) Genomic annotation and distribution of non-telomeric TRF2-G4 candidate regulatory regions identified in MuSCs.

Supplementary Fig. 16

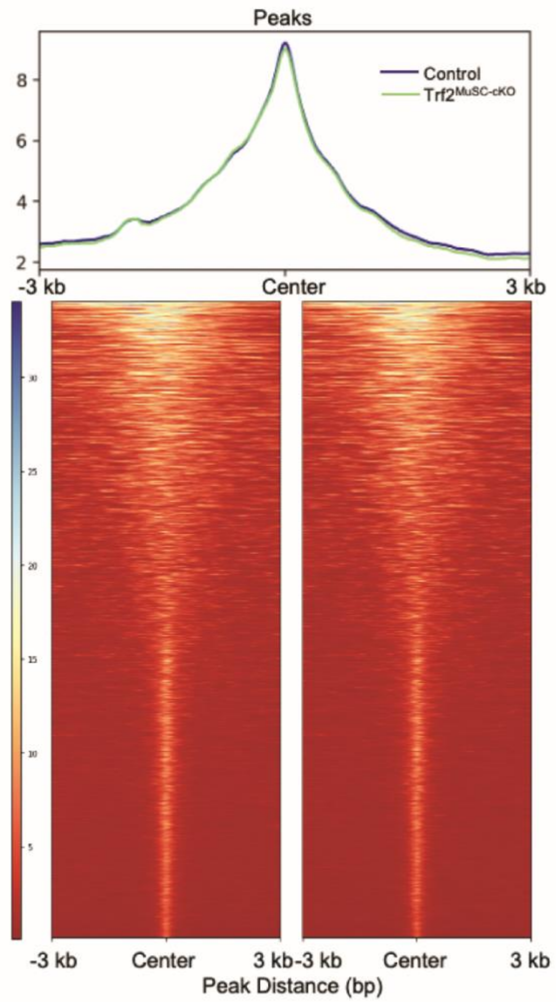

Fig. S16. Chromatin accessibility is largely preserved following TRF2 deletion in MuSCs. Heatmap of ATAC-Seq signal at peaks linked to genes differentially expressed following TRF2 deletion in MuSCs, comparing control and $TRF2^{MuSC\text{-}cKO}$ conditions. Peaks are ranked by decreasing signal intensity in control samples and displayed in the same order across both genotypes.

Supplementary Fig. 17

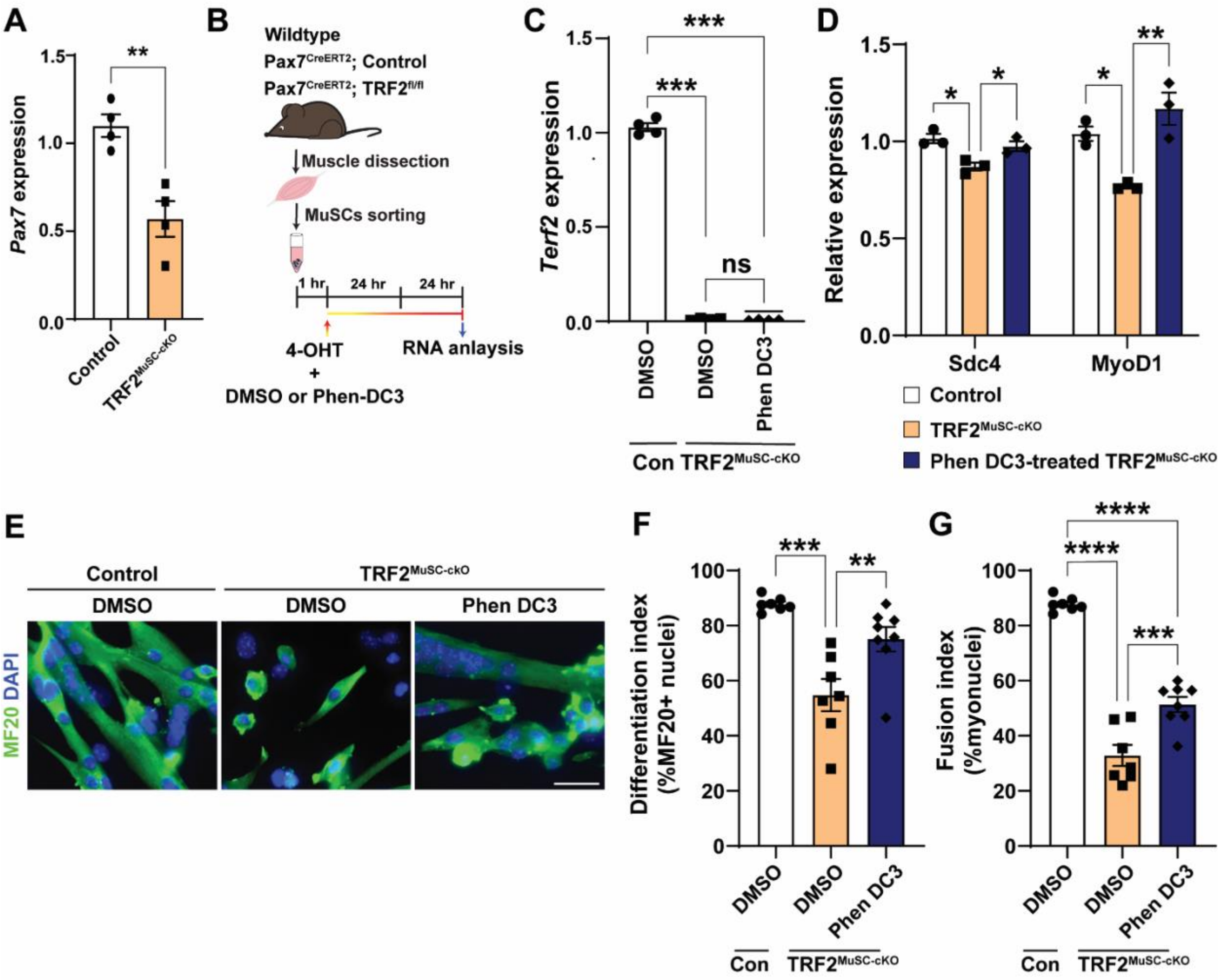

Fig. S17. G-quadruplex stabilization restores stem cell gene expression and mitigates defective myogenesis in TRF2-deficient MuSCs.

(A) Quantitative RT-PCR analysis of *Pax7* expression in control and TRF2$^{MuSC\text{-}cKO}$ MuSCs (n=4 mice per genotype, 3 months old). **(B)** Experimental design: MuSCs were isolated from wild-type, control, and TRF2$^{MuSC\text{-}cKO}$ muscles, treated with 4-hydroxytamoxifen (4-OHT) to induce TRF2 deletion, and subsequently exposed to either DMSO (vehicle control) or PhenDC3 (2µM), a G4-stabilizing compound. **(C)** Quantitative RT-PCR analysis of *Terf2* expression in controls and TRF2$^{MuSC\text{-}cKO}$ MuSCs treated with DMSO or PhenDC3 (2µM). Note that G4 stabilization does not affect *Terf2* expression in TRF2$^{MuSC\text{-}cKO}$ MuSCs (n=4 mice, 3-month-old, per genotype). (**D**) Quantitative RT-PCR analysis of *Sdc4* and *MyoD1* expression in control and TRF2$^{MuSC\text{-}cKO}$ MuSCs treated with PhenDC3 (2µM). MuSCs were isolated from n=3 mice (3 months old) per genotype and condition. (**E**) Representative images of differentiated control (DMSO treated) and TRF2$^{MuSC\text{-}cKO}$ cells treated either with DMSO or PhenDC3 stained for the differentiation marker myosin heavy chain (MF20) and nuclei (DAPI). Scale bar, 20µm. **(F)** Differentiation index, defined as the percentage of MF20 positive nuclei relative to total nuclei. **(G)** Fusion index, defined as the percentage of nuclei within MF20 positive myotubes containing 3 or more nuclei divided by the

total number of nuclei. n=7 biological replicates per genotype and condition. Data are presented as means ±SEM. Statistical analysis was performed using two-tailed unpaired t-tests with Welch's correction in (A) and one-way ANOVA with Tukey's multiple-comparison test in (C-D and F-G). *P<0.05; **P< 0.01; p<0.001; ****P<0.0001; ns, non-significant.

Supplementary Fig. 18

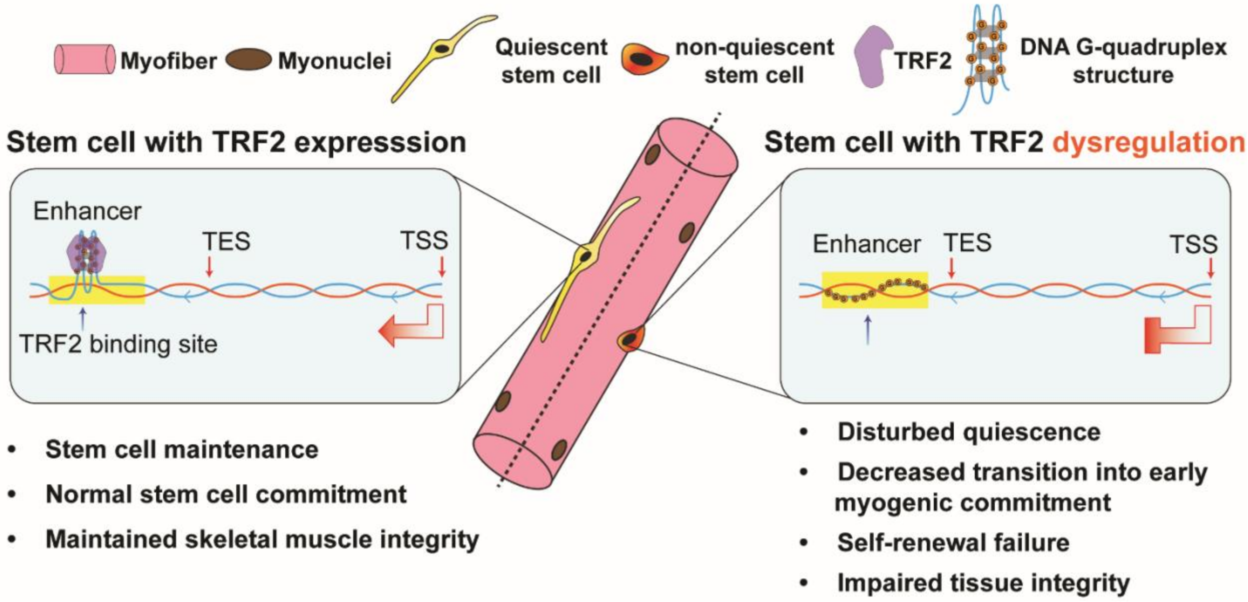

Fig S18. Proposed model linking TRF2-mediated G4 regulation to muscle stem cell function.

Schematic summarizing the mechanism of action of TRF2 in muscle stem cell (MuSC) maintenance and regeneration. In quiescence MuSCs, high TRF2 expression leads to binding on G4 structures within the regulatory regions of pivotal stem cell genes, including *Pax7,* in order to maintain stem cell identity that is critical for high regeneration capacity and self-renewal. In case of TRF2 reduction, TRF2-G4s association are not maintained, MuSCs lose their ability to maintain stemness. While upon injury these cells proliferate, they are unable to move forward into myogenic commitment and fail to self-renew, resulting in impaired function, and severe regeneration defects.

Table S1. Antibodies used in this study.

Antibodies	Suppliers	Catalog numbers and identifiers
Anti-Alpha 7 integrin Alexa Fluor 647, Clone R2F2	ablab.ca	Cat# 67-0010-05
Alex flour 555 Goat anti-rabbit IgG	Thermo Fisher Scientific	Cat# A-21428 RRID: AB_2535849
Alex flour 648 Goat anti-rat IgG	Thermo Fisher Scientific	Cat# A-21247 RRID: AB_141778
Alexa flour 488 Goat anti-mouse IgG1	Thermo Fisher Scientific	Cat# A-21121 RRID: AB_2535764
Alexa flour 488 Goat anti-rabbit IgG	Thermo Fisher Scientific	Cat# A32731 RRID: AB_2633280
Mouse anti-MYH3 antibody (F1.652)	DSHB	Cat# F1.652 RRID: AB_528358
Mouse anti-Pax7	DSHB	Cat# Pax7 RRID: AB_2299243
Mouse anti-MyoG	DSHB	Cat# F5B RRID: AB_2146602
Mouse anti-Phospho-ATM (Ser1981) Antibody	Thermo Fisher Scientific	Cat# MA1-2020 RRID: AB_1086244
Rabbit anti-53bp1	Novus Biologicals	Cat# NB100-304 RRID: AB_10003037
Rabbit anti-Phospho Histone H2A.X (Ser139) (20E3)	Cell Signaling Technology	Cat# 9718 RRID: AB_2118009
Rabbit anti-TRF-2	Novus Biologicals	Cat# NB100-57130 RRID: AB_844199
Rabbit anti-Perilipin-1 (D1D8)	Cell Signaling Technology	Cat# 9349 RRID: AB_10829911
Rat anti-CD11b-biotin, Clone M1/70	BD Biosciences	Cat# 553309; RRID: AB_394773
Rat anti-CD31-biotin	Fisher Scientific	Cat# 13-0311-85; RRID: AB_466421
Rat anti-CD45-biotin	BD Biosciences	Cat# 553078; RRID: AB_394608
Rat anti-Laminin B2, Clone A5	Fisher Scientific	Cat# 05-206-MI RRID: AB_309655
Rat anti-Ly-6A/E-biotin (Sca1), clone E13-161.7	BD Biosciences	Cat# 553334; RRID: AB_394790

Rat anti-mouse CD106-Alexa flour 647, Colne 429(MVCAM.A)	BD Biosciences	Cat# 561612<br>RRID: AB_10896662
Rat anti-mouse CD34-BV421, Clone RAM34	BD Biosciences	Cat# 562608;<br>RRID: AB_11154576

Table S2. Primers used in this study.

Primers	Suppliers and assay ID
Cdkn2a(p16)-FAM	Thermo Fisher Scientific, Mm00494449_m1
Cdkn2c(p57)-FAM	Thermo Fisher Scientific, Mm00438170_m1
PAX7-FAM	Thermo Fisher Scientific, Mm00834079_m1
TERF2-FAM	Thermo Fisher Scientific, Mm01253555_m1
Trp53(p53)-FAM	Thermo Fisher Scientific, Mm01731290_g1
TBP-VIC-MGB_PL	Thermo Fisher Scientific, Mm01277042_m1
Gapdh-VIC-MGB	Thermo Fisher Scientific, Cat# 4352339e
